# Sea Anemone Kunitz Peptide HCIQ2c1: Structure, Modulation of TRPA1 Channel, and Suppression of Nociceptive Reaction In Vivo

**DOI:** 10.3390/md22120542

**Published:** 2024-12-02

**Authors:** Aleksandra N. Kvetkina, Sergey D. Oreshkov, Pavel A. Mironov, Maxim M. Zaigraev, Anna A. Klimovich, Yulia V. Deriavko, Aleksandr S. Menshov, Dmitrii S. Kulbatskii, Yulia A. Logashina, Yaroslav A. Andreev, Anton O. Chugunov, Mikhail P. Kirpichnikov, Ekaterina N. Lyukmanova, Elena V. Leychenko, Zakhar O. Shenkarev

**Affiliations:** 1 G.B. Elyakov Pacific Institute of Bioorganic Chemistry, Far Eastern Branch, Russian Academy of Sciences, 690022 Vladivostok, Russia; kvetkinaan@gmail.com (A.N.K.); annaklim_1991@mail.ru (A.A.K.); yliya77ya@mail.ru (Y.V.D.); almenshov1990@gmail.com (A.S.M.); leychenko@gmail.com (E.V.L.); 2 Shemyakin-Ovchinnikov Institute of Bioorganic Chemistry, Russian Academy of Sciences, 119997 Moscow, Russia; seryynut@gmail.com (S.D.O.); mironov@nmr.ru (P.A.M.); maximzaigraev@yandex.ru (M.M.Z.); d.kulbatskiy@gmail.com (D.S.K.); yulia.logashina@gmail.com (Y.A.L.); aya@ibch.ru (Y.A.A.); anton.chugunov@gmail.com (A.O.C.); lyukmanova_ekaterina@smbu.edu.cn (E.N.L.); 3 Moscow Center for Advanced Studies, 123592 Moscow, Russia; 4 Interdisciplinary Scientific and Educational School of Moscow University «Molecular Technologies of the Living Systems and Synthetic Biology», Faculty of Biology, Lomonosov Moscow State University, 119234 Moscow, Russia; 5 Shenzhen MSU-BIT University, No. 1, International University Park Road, Dayun New Town, Longgang District, Shenzhen 518172, China

**Keywords:** Kunitz-peptide, sea anemone, TRPA1, pain, nociceptive reaction, in vivo, capsaicin, AITC

## Abstract

TRPA1 is a homotetrameric non-selective calcium-permeable channel. It contributes to chemical and temperature sensitivity, acute pain sensation, and development of inflammation. HCIQ2c1 is a peptide from the sea anemone *Heteractis magnifica* that inhibits serine proteases. Here, we showed that HCIQ2c1 significantly reduces AITC- and capsaicin-induced pain and inflammation in mice. Electrophysiology recordings in *Xenopus* oocytes expressing rat TRPA1 channel revealed that HCIQ2c1 binds to open TRPA1 and prevents its transition to closed and inhibitor-insensitive ‘hyperactivated’ states. NMR study of the ^15^N-labeled recombinant HCIQ2c1 analog described a classical Kunitz-type structure and revealed two dynamic hot-spots (loops responsible for protease binding and regions near the *N*- and *C*-termini) that exhibit simultaneous mobility on two timescales (ps–ns and μs–ms). In modelled HCIQ2c1/TRPA1 complex, the peptide interacts simultaneously with one voltage-sensing-like domain and two pore domain fragments from different channel’s subunits, and with lipid molecules. The model explains stabilization of the channel in the open conformation and the restriction of ‘hyperactivation’, which are probably responsible for the observed analgetic activity. HCIQ2c1 is the third peptide ligand of TRPA1 from sea anemones and the first Kunitz-type ligand of this channel. HCIQ2c1 is a prototype of efficient analgesic and anti-inflammatory drugs.

## 1. Introduction

The transient receptor potential ankyrin 1 (TRPA1) is a calcium-permeable channel belonging to the Transient Receptor Potential (TRP) ion channel family, which in turn belongs to the large superfamily of P-loop cation channels [1,2]. Like all TRP channels, TRPA1 forms homo-tetramers with each subunit composed of six transmembrane (TM) helices (S1–S6) and cytoplasmic *N*- and *C*-termini. Within the membrane, TRPA1 consists of several modules: four identical distal voltage-sensing-like domains (VSLDs), each formed by four TM helices (S1–S4); and a central pore domain (PD) composed by helices S5 and S6 from four subunits. Between the helices S5 and S6 there is a membrane reentrant P-loop containing two short helices P1 and P2 [3]. TRPA1 has a “domain swapped” topology where the VSLD of one subunit contacts the S5–S6 helices of the previous subunit (numbered counterclockwise when viewed from the extracellular side). The elongated *N*- and *C*-terminal fragments of the TRPA1 subunits form cytoplasmic coupling and ankyrin repeats domains [3]. The latter ones contain 14–18 ankyrin repeats and are involved in protein–protein interactions, coupling with other TM proteins and cytoskeleton, and transport of the channel to the plasma membrane [4]. The cytoplasmic coupling domains contain several free cysteine residues responsible for the channel activation by endogenous mediators and exogenous ligands, which covalently bind to these residues [5,6]. The binding sites of non-covalent ligands are located in the pore domain where antagonists bind [3,6], at the interface between the VSLD, pore, and cytoplasmic domains where both agonists and antagonists bind [7,8,9], and at the extracellular interface of VSLD where spider inhibitory toxins bind [10].

TRPA1 is widely distributed in primary sensory neurons, epithelial cells of intestines, lungs, bladder, and the inner ear cells of humans and animals, as well as in macrophages, dendritic cells, T-lymphocytes, neutrophils, and mast cells [11,12,13,14]. This channel is involved in the transmission of nociceptive signals, including external mechanical impact, temperature changes, chemical irritation that induces acute pain, and is also activated by endogenous ligands formed during inflammation, which is often accompanied by chronic pain. Therefore, TRPA1 is considered important target for novel analgesic and anti-inflammatory drugs. Interestingly, in native sensory neurons, the TRPA1 channel can interact with another nociceptive TRP channel, TRPV1, allowing adaptation of the biophysical properties of the single channels [15].

TRPA1-induced pain can be inhibited by both natural and synthetic compounds. Among the selective antagonists, only five have been tested in clinical trials for the treatment of pain or other pathologies [16]. GRC 17536 (Glenmark Pharmaceuticals) and HX-100 (Hydra Biosciences in partnership with Cubist Pharmaceuticals) were effective in painful diabetic neuropathy. CB-189625 (Hydra Biosciences in partnership with Cubist Pharmaceuticals) was used for acute surgical pain, but the study was terminated due to disappointing pharmacokinetics. ODM-108 (Orion Pharma) was targeted for neuropathic pain but had poor pharmacodynamic properties. GDC-0334 (Genentech/Roche) was applied for the treatment of asthma and tested in a phase 1 trial, but it was not further developed [16].

Sea anemones are a rich source of biologically active peptides acting on diverse ion channels [17,18], however, data on TRPA1 channel ligands from sea anemones are limited. In 2017, two peptides, Ms 9a-1 (35 a.a.) and Ueq 12-1 (45 a.a.), were isolated from *Metridium senile* and *Urticina eques,* respectively, and have been shown to be positive modulators of TRPA1 and produce analgesic and anti-inflammatory effects in vivo [19,20].

Here we report a third peptide ligand of the TRPA1 channel from sea anemones, the HCIQ2c1 (58 a.a.). It is a representative of a combinatorial library of Kunitz-peptides from *Heteractis magnifica* [21]. (This anemone initially was erroneously classified as *Heteractis crispa*.) Traditionally, Kunitz-peptides are efficient inhibitors of serine proteases [21]. In addition, HCIQ2c1 exhibits a pronounced cytoprotective effect on neuroblastoma cells, although its molecular target(s) was not identified yet [21,22]. HCIQ2c1 also suppresses the ATP-induced production of reactive oxygen species (ROS) and interacts with the extracellular domain of P2X purinoceptor 7 (P2X7R) [22]. Electrophysiological experiments revealed that HCIQ2c1 does not influence the TRPV1 channel activity [22].

Here, we demonstrated the antinociceptive activity of HCIQ2c1 in several in vivo mouse models of acute pain and inflammation. Based on the results of electrophysiology experiments on the rat TRPA1 channel and NMR study of HCIQ2c1 in solution, we modelled the HCIQ2c1 complex with open TRPA1 in a lipid membrane using a combined ensemble protein–protein docking/molecular dynamics (MD) protocol. The observed mode of TRPA1/HCIQ2c1 interaction suggests that the peptide restricts the mutual motions of the channel domains and thus stabilizes it in the open conformation.

## 2. Results

### 2.1. Expression and Purification of HCIQ2c1 and ^15^N-HCIQ2c1 Analogue

To study the structure and functional properties of the HCIQ2c1 peptide, its recombinant unlabeled and ^15^N-labeled variants were produced using the *Escherichia coli* expression system. The TRX-HCIQ2c1 fusion proteins were purified on Ni^2+^-NTA agarose, followed by CNBr cleavage after the Met residue placed upstream of the first residue of the HCIQ2c1 sequence. The target peptides were purified by RP-HPLC. The retention time of ^15^N-HCIQ2c1 on a reverse-phase column was increased by 1.5 min compared to the unlabeled peptide (37.0 min and 35.5 min, respectively, Figure S1) [21]. The final yields were 10 mg and 0.5 mg from 1 L of cell culture for the unlabeled and ^15^N-labeled peptide, respectively. According to Matrix-Assisted Laser Desorption/Ionization Time-of-Flight Mass Spectrometry (MALDI TOF/MS) data, the molecular mass of ^15^N-HCIQ2c1 was increased by 60 Da relative to the unlabeled peptide (6390 Da and 6330 Da, respectively, Figure S1, insert), indicating a 72.3% degree of ^15^N labeling.

### 2.2. HCIQ2c1 Does Not Influence Motor and Orienting-Exploratory Activities in the Open Field Test

Adult female CD-1 mice were used for behavioral experiments and pain tests. Despite the reported difference in pain sensitivity between male and female rats [23], we note that recent work has shown that sex difference in mice does not significantly influence the commonly used behavioral tests, including the Open field and Hot plate tests [24]. Therefore, we assume that the results of the studies will not differ significantly depending on the sex of the mice.

We estimated whether HCIQ2c1 influences motor and orienting-exploratory activities of mice in the Open field test. The administration of the peptide in the quadriceps muscle of the left hind paw at doses of 0.01, 0.1, and 1 mg/kg 60 min before the testing did not have any noticeable effect on the mice behavior in the Open field test compared to the control group of animals (treated with saline). A non-significant decrease in overall activity time and the number of rearing up was observed in the animal groups treated with HCIQ2c1 compared to the control group (Table S1). These data indicate that HCIQ2c1 does not exhibit neurotrophic or neurotoxic effects and negative influence on locomotor system of the animals at the doses up to 1 mg/kg. Thus, the efficacy of HCIQ2c1 in the pain models (see below) did not result from locomotor impairment or sedation.

### 2.3. HCIQ2c1 Influences Acute Pain Sensibility in the Hot Plate Test

**Figure 1:**
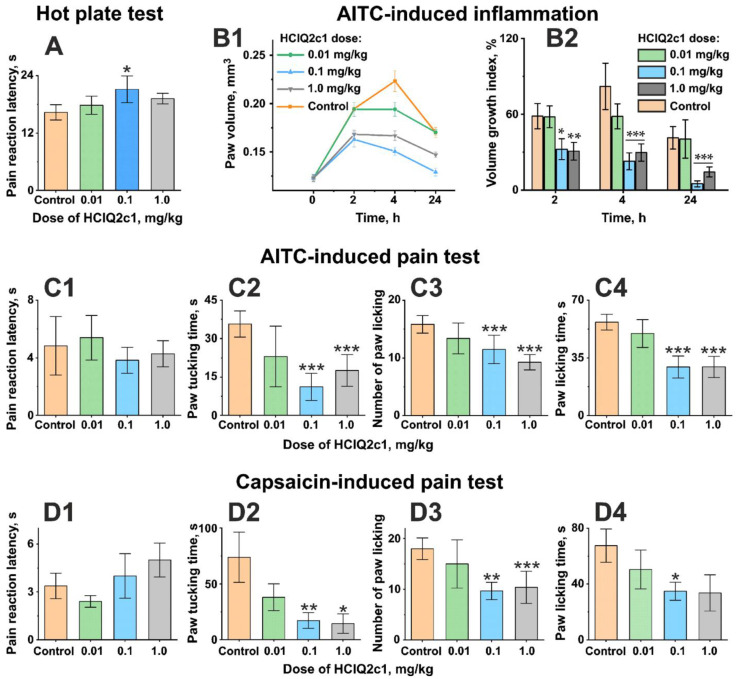
Analgesic activity of HCIQ2c1 in vivo. (**A**) The pain threshold in the Hot plate test was detected as latency to withdraw or lick the fore or hind paw. (**B**) Time-dependent effect of HCIQ2c1 on the volume of paw subcutaneous injected with 0.05% AITC (**B1**) and Volume Growth Index (%) (**B2**). (**C**) Analgesic activity of HCIQ2c1 in a model where pain was induced by subplantar injection of 0.05% AITC. (**D**) Analgesic activity of HCIQ2c1 in a model where pain was induced by subplantar injection of 6 µg/mouse capsaicin. The pain threshold was detected as: (**C1**,**D1**) latency to pain-related response or nociceptive behavior (first licking, tucking, scratching, flicking, or biting the injected hind paw), (**C2**,**D2**) time spent tucking the injected paw, (**C3**,**D3**) the number of licking the injected paw, and (**C4**,**D4**) time spent licking. HCIQ2c1 or saline buffer (control) was administrated intramuscularly 60 min before start of the test (**A**), or AITC (**B**,**C**) or capsaicin (**D**) injection. Data are presented as mean ± S.E.M. (*n* = 7). * *p* < 0.05, ** *p* < 0.01, and *** *p* < 0.001 indicate significant differences between the control and HCIQ2c1 groups according to one-way ANOVA/Dunnett’s multiple comparisons test.

The analgesic activity of HCIQ2c1 was studied using the Hot plate test, in which acute pain was induced by placing mice on the plate heated to 52 °C. The pain sensibility was assessed as latency of withdrawal or licking of the fore or hind paws (in seconds). A significant increase in the pain response latency (from 16.3 ± 1.0 to 21.8 ± 1.7 s) was observed in the animal group receiving intramuscularly 0.1 mg/kg HCIQ2c1 (Figure 1A).

### 2.4. Analgesic and Anti-Inflammatory Activities of HCIQ2c1 in the AITC-Induced Nociceptive Behavior Test

Allyl-isothiocyanate (AITC), one of the TRPA1 channel covalent agonists, exhibits a strong local irritant effect accompanied by pain, lacrimation, hyperemia, itching, and edema. The AITC-induced pain sensitivity and inflammation test are widely used to study the TRPA1 channel modulators involved in pain signal transduction and nociception [25,26]. To assay both analgesic and anti-inflammatory potential of HCIQ2c1, AITC was injected into the plantar skin of the hind paw pad to provoke pain behavior associated with the TRPA1 activation. Intramuscular administration of HCIQ2c1 in mice before AITC did not change the pain reaction latency (Figure 1C1). At the same time, the peptide at doses of 0.1 and 1 mg/kg significantly reduced the time of paw tucking by 3 and 2 times, respectively (Figure 1C2), as well as reduced the number and time of paw licking by more than 1.5 times compared to the control group (Figure 1C3,C4).

The anti-inflammatory activity of HCIQ2c1 was assessed by the peptide’s ability to suppress swelling and edema of paw into which AITC was injected (Figure 1B). In the control group, the paw volume increased significantly and became larger by ~60% and ~80% during 2 h and 4 h after AITC subcutaneous injection, respectively (Figure 1B1). At 24 h after AITC injection, the paw volume decreased but did not reach the initial value (volume growth index ~35%). In contrast, intramuscular administration of HCIQ2c1 at doses 0.1 and 1.0 mg/kg significantly suppressed the paw swelling by 1.5-fold within 2 h and by more than 3.5-fold within 4 h after the AITC application. The paw size was almost completely restored after 24 h (volume growth index ~10%, Figure 1B2). HCIQ2c1 at the 0.01 mg/kg dose exhibited slight insignificant activity, reaching a maximum at 4 h after the AITC injection. Thus, HCIQ2c1 at the doses of 0.1 mg/kg and higher effectively suppresses pain sensitivity and AITC-induced inflammation.

### 2.5. Analgesic Activity of HCIQ2c1 in the Capsaicin-Induced Pain Test

Capsaicin is the spicy ingredient of the hot red chili pepper and a natural agonist of the TRPV1 channels. TRPV1 is involved in the modulation of nociceptive inputs to the spinal cord and brain stem centers, in the integration of diverse painful stimuli and in somatic and visceral peripheral inflammation [27]. In the sensory neurons, TRPA1 and TRPV1 channels are coupled, influencing each other’s responses to various pain-inducing signals [15]. Intradermal capsaicin injection contributes to the development of thermal and mechanical hyperalgesia.

To evaluate the analgesic activity of HCIQ2c1 in the capsaicin-induced pain test, capsaicin was injected under the plantar skin of the hind paw pad, and the following parameters were measured: (1) latency of the pain-related response, (2) time spent in tucking the injected paw, (3, 4) number and time of licking the injected paw (Figure 1D). Intramuscular injection of HCIQ2c1 1 h before capsaicin at the doses of 0.1 and 1.0 mg/kg increased the latency of nociceptive behavior and reduced other effects compared to the control group of mice. In the most cases, these effects were statistically significant (Figure 1D). Interestingly, the peptide at the lower dose of 0.01 mg/kg slightly reduced the time of paw tucking and licking, but these changes were not significant (Figure 1D2,D4).

### 2.6. HCIQ2c1 Is an Allosteric Modulator of Rat TRPA1

The results of behavioral tests point to TRPA1 as one of the possible targets of HCIQ2c1. To test this hypothesis, we examined the effects of recombinant HCIQ2c1 in *Xenopus laevis* oocytes expressing rat TRPA1 channel using the two-electrode voltage-clamp method. We used fast voltage ramps from −80 mV to +80 mV to record inward and outward currents in a single experiment [19,20,28]. Currents were elicited by the non-covalent agonist diclofenac or the covalent agonist AITC. Preliminarily experiments showed that the effects of HCIQ2c1 at concentrations 10 and 100 µM were similar in amplitude. Therefore, concentration of 10 µM HCIQ2c1 was used in all subsequent experiments. Application of HCIQ2c1 itself did not induce any currents and changes in leakage currents.

**Figure 2:**
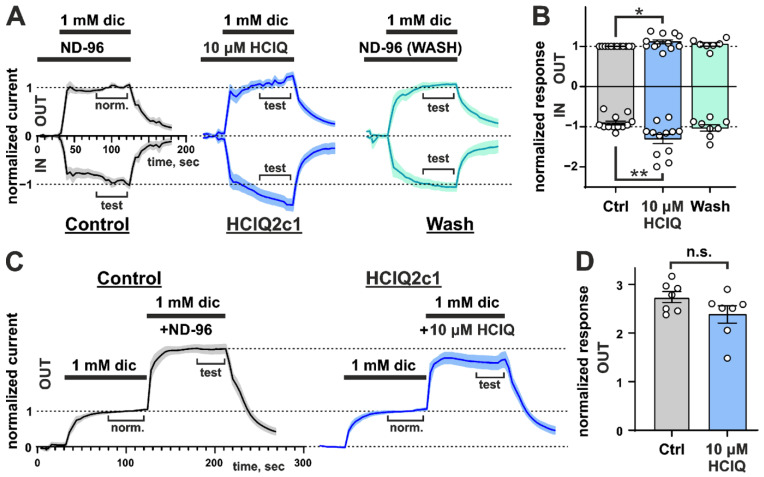
Recombinant HCIQ2c1 affects the diclofenac-evoked currents in *X. laevis* oocytes expressing rat TRPA1 in the experiment with 30-s HCIQ2c1 preincubation (**A**,**B**) and does not affect the currents in the experiment with 90-s pre-pulse of the agonist and second simultaneous 90-s HCIQ2c1+diclofenac pulse (**C**,**D**). (**A**,**C**) Average current traces normalized to the amplitude of the currents in the time interval labelled “norm.” (*n* = 12 (**A**), *n* = 6–7 (**C**), different oocytes were recorded, S.E.M. range is shown as the shade around the trace). Three (**A**) or two (**C**) consecutive responses (Control, HCIQ2c1, Wash) were measured on each oocyte at 5 min intervals. Direction of the current is shown by the labels “OUT” and “IN”. The application of compounds is shown by bars above the current traces. The amplitude of responses was measured at time points labeled “test”. The concentrations of diclofenac and HCIQ2c1 were 1 mM and 10 µM, respectively. (**B**,**D**) The normalized current amplitudes (mean ± S.E.M.). n.s., not significant. * *p* < 0.05 and ** *p* < 0.01 indicate significant differences between the “HCIQ2c1” and “Control” data groups with the same direction of currents based on one-sample (**B**, OUT) and two-sample (**B**, IN) two-sided *t*-tests. No significant differences were found for the data presented in panel (**D**). The non-normalized current traces for the data presented in this figure are shown in Figure S2.

In studies with diclofenac stimulation, HCIQ2c1 was applied either during a 30-s pre-incubation before current stimulation with the diclofenac+HCIQ2c1 mixture (Figure 2A) or after a 90-s agonist pre-pulse, also simultaneously with diclofenac (Figure 2C). Pre-incubation with 10 µM HCIQ2c1 resulted in significant increase in the amplitude of both outward and inward diclofenac-evoked currents by ~11% and ~44%, respectively (*p* < 0.05 and <0.01, respectively, Figure 2B). The increase in amplitude was abolished upon HCIQ2c1 wash-out, suggesting a reversible HCIQ2c1 interaction outside the diclofenac binding pocket. At the same time, application of the peptide after initial stimulation of oocytes with a pre-pulse of diclofenac, during the second agonist pulse, slightly reduced the amplitude of the outward currents by ~13%, but this effect was not statistically significant (Figure 2D).

**Figure 3:**
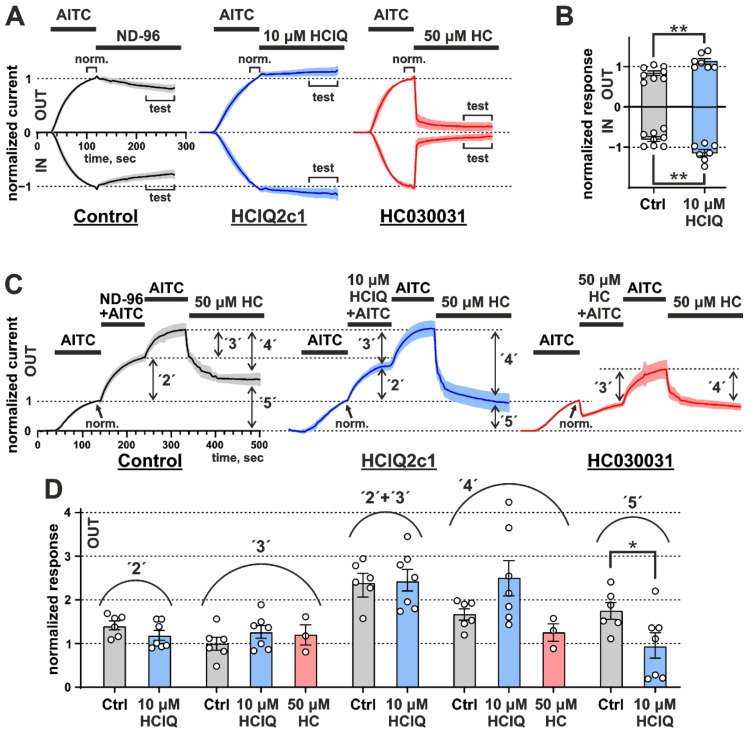
Recombinant HCIQ2c1 affects the residual currents through rat TRPA1 in *X. laevis* oocytes after 90-s AITC pulse (**A**,**B**) and does not affect the currents in the experiment with 100-s AITC pre-pulse, the second simultaneous 100-s HCIQ2c1+AITC pulse, the third ‘readout’ 100-s AITC pulse, and the final application of the antagonist HC030031 (**C**,**D**). (**A**) Average current traces normalized to the amplitude of the currents in the time interval labelled “norm.” (*n* = 7–8 (**A**), *n* = 6–7 (**C**), each response was measured on a distinct oocyte, S.E.M. range is shown as the shade around the trace). Direction of the current is shown by the labels “OUT” and “IN”. The application of compounds is shown by bars above the current traces. The amplitude of responses was measured at time points labeled “test” or marked with arrows. The TRPA1 antagonist HC030031 was used as a negative control. The concentrations of AITC, HCIQ2c1, and HC030031 were 100 µM, 10 µM, and 50 µM, respectively. (**B**,**D**) The normalized current amplitudes (mean ± S.E.M.). * *p* < 0.05 and ** *p* < 0.01 indicate significant differences between the “HCIQ2c1” and “Control” data groups with the same direction of currents based on two-sided *t*-tests. The only significant difference in panel (**D**) is the difference in residual current level after application of HC030031. The non-normalized current traces for the data presented in this figure are shown in Figure S3.

In the experiments with AITC stimulation, HCIQ2c1 was applied either alone after the agonist pulse (Figure 3A) or simultaneously with the second AITC pulse as the part of the train containing three agonist pulses (Figure 3C). In the former case, peptide application resulted in the significant increase in the sustained component of the outward and inward currents by ~37% and ~45%, respectively (*p* < 0.01, Figure 3B), indicating the ability of the peptide to either impede the agonist dissociation or to reduce the rate of the channel desensitization or transition from the open to the closed state.

Application of HCIQ2c1 together with the second AITC pulse resulted in the insignificant decrease in normalized response by ~16% compared to the control, where no HCIQ2c1 was applied (Figure 3C,D, experimental point ‘2’). Interestingly, this decrease in the response was compensated by the third application of AITC (Figure 3C,D, experimental point ‘3’). The sum of the normalized responses (‘2 + 3’) was independent from HCIQ2c1 application. To check the state of the oocyte membrane and TRPA1 channels after three pulses of AITC, 50 µM of the TRPA1 inhibitor HC030031 was applied at the end of the experimental protocol. In control experiments (three pulses of pure AITC), the significant fraction of open channels (~ 50%) became insensitive to the inhibitor application (Figure 3C,D, experimental point ‘5’). Increase in the HC030031 concentration or its additional application did not alter the channels behavior. Probably, the prolonged stimulation switched some fraction of the channels to the state (we can call it ‘hyperactivated’) that is not sensitive to the action of antagonist HC030031. On the other hand, application of HCIQ2c1 during the second agonist pulse significantly increased the efficiency of the added inhibitor by reducing the fraction of these ‘hyperactivated’ channels to ~30% (*p* < 0.05, Figure 3C,D, experimental point ‘5’).

Taken together, the observed effects are consistent with the allosteric ‘normalizing’ activity of HCIQ2c1 on the TRPA1 channel. Experiments with HCIQ2c1 pre-incubation (Figure 2A) and sustained currents (Figure 3A) suggest that the peptide can bind to the TRPA1 channel in the open conformation (and probably in the closed conformation, too) and impedes the dissociation of non-covalent and covalent agonists or slows down the channel desensitization. On the other hand, under prolonged or repeated stimulation with the covalent agonist AITC, HCIQ2c1 probably limits the TRPA1 activation and protects the channel from switching to the inhibitor-insensitive ‘hyperactivated’ state. This mode of action also requires interaction of the peptide with the open conformation of TRPA1. This restriction of ‘hyperactivation’ is likely responsible for the analgesic activity of HCIQ2c1 observed in vivo (see above).

### 2.7. Kunitz-Type 3D Structure of HCIQ2c1

**Figure 4:**
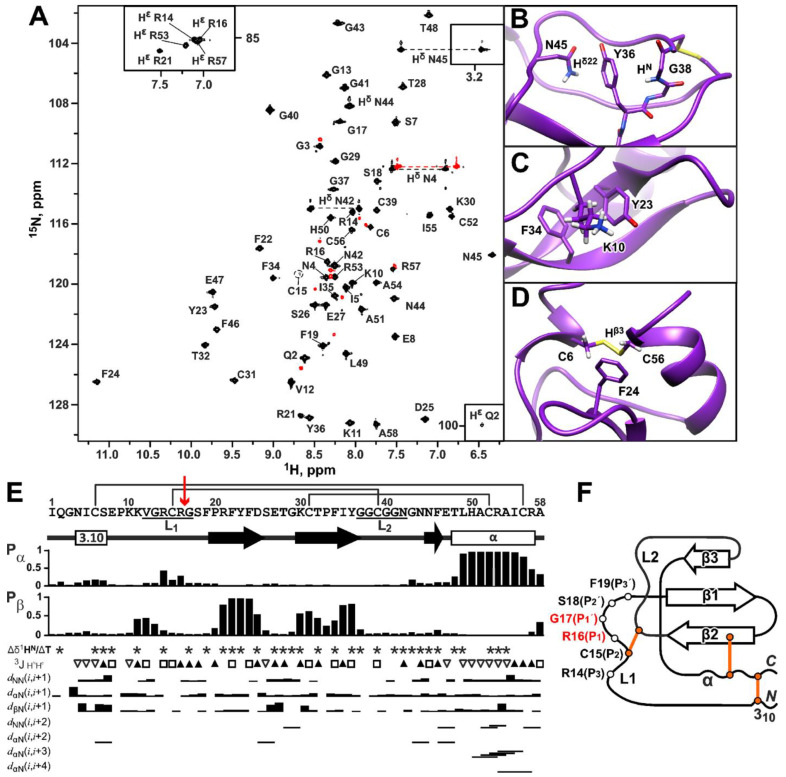
NMR data define the HCIQ2c1 secondary structure. (**A**) 2D ^15^N-HSQC spectrum of 0.08 mM ^15^N-labeled HCIQ2c1 (30 °C, pH 4.5). The resonances of side chain NH_2_ groups are connected by dashed lines. The system of minor signals is shown in red color. (**B**–**D**) The ring-current contributions from the nearby aromatic side chains explain atypical up–field shifts of ^1^H^δ22^ Asn45 and ^1^H^N^ Gly38 resonances (**B**), ^1^H_2_C^β^ and ^1^H_2_C^γ^ resonances of the Lys10 side chain (**C**), and ^1^H^β3^ resonance of Cys56 (**D**). The secondary structure of HCIQ2c1 (**E**). Elements of the secondary structure were calculated using the STRIDE program [29] from the determined spatial structure of HCIQ2c1 (see below). The β-strands are designated by arrows, α- and 3_10_ helices by rectangles. The L_1_ and L_2_ loops are underlined. Possible position of the protease cleavage site is shown by red arrow. The probabilities of the residues to participate in the α-helix or β-strand (P_α_ and P_β_) were calculated from the chemical shifts in the TALOS-N software [30]. Asterisks indicate the residues with low amplitude of the amide proton temperature gradient (|Δδ^1^H^N^/ΔT| < 4.5 ppb/°K). Small (<6 Hz), large (>8 Hz), and medium (others) ^3^J_H_^N^_H_^α^ coupling constants are indicated by empty, filled triangles, and open squares, respectively. Map of NOE contacts (τ_m_ = 100 ms) is shown as usual. (**F**) Topology of the HCIQ2c1 secondary structure. The residues possibly forming a protease binding site are marked.

The measured 2D ^15^N-HSQC spectrum of ^15^N-labeled HCIQ2c1 revealed a high resonance dispersion typical for folded β-structural proteins (Figure 4A). The combination of 2D/3D TOCSY and NOESY spectra with the 2D ^13^C-HSQC spectrum measured at a natural isotopic abundance provided an almost complete assignment of the ^1^H, ^15^N, and ^13^C resonances of HCIQ2c1. Signals of all HCIQ2c1 residues were observed in the NMR spectra. However, the ^1^H-^15^N cross-peak of Cys15 was significantly broadened (Figure 4A, encircled) and the ^1^H-^15^N cross-peak of Gly38 was not detected in the 2D ^15^N-HSQC spectrum. The ^1^H^N^ resonance of Gly38 was assigned using H^N^_i_–H^α^_i+1_ contact in the 2D NOESY spectrum. This broadening indicates the presence of an intermediate (on the NMR timescale) conformational exchange process occurring in the HCIQ2c1 structure around the Cys15-Cys39 disulfide bond.

The configurations of the Xxx–Pro peptide bonds were determined from the ^13^C chemical shifts of Pro residues observed in the 2D ^13^C-HSQC spectrum. The δ[^13^C_β_]–δ[^13^C_γ_] differences for Pro9, Pro20, and Pro33 (4.78, 3.87, and 5.25 ppm, respectively) were in the typical range for *trans*-Xxx–Pro dipeptides [31]. In the cases of Phe19–Pro20 and Thr32–Pro33 dipeptides, this configuration was confirmed by the observation of strong H^α^_i_–H^δ^_i+1_ cross-peaks in the 2D NOESY spectra. At the same time, the presence or absence of H^α^_i_–H^δ^_i+1_ or H^α^_i_–H^α^_i+1_ contacts for the Glu8–Pro9 dipeptide could not be established due to overlap with the residual water signal. Interestingly, a system of additional minor NMR signals with a relative abundance of ~10% was observed for the *N*- and *C*-terminal residues (Gln2–Cys6, Arg53, Cys56, Arg57, Figure 4A, red), suggesting the presence of slow (on the NMR timescale) fluctuations in the conformation of the Cys6–Cys56 disulfide bond, which may be accompanied (or even induced) by *cis-trans* isomerization of the Glu8–Pro9 bond. The minimal difference in the resonance frequencies of the signals of the two conformers (15 Hz) was observed for ^1^H^N^ resonance of Arg57. This indicates that the corresponding conformational exchange process has a time scale significantly greater than 70 ms, but it is not known whether this exchange is related to conformational changes around the Cys15–C39 bond. Nevertheless, we conclude that the proline residues in the major HCIQ2c1 isoform have a *trans*–configuration.

On the other hand, the core of the peptide was well-ordered, so atypical chemical shifts were observed due to ring-current shifts from the nearby aromatic side chains with fixed orientations. The ^1^H^δ22^ resonance of Asn45 and ^1^H^N^ signal of Gly38 were shifted up–field to 3.2 and 4.3 ppm, respectively, due to interaction with the aromatic group of Tyr36 (Figure 4B). Interestingly, similar up–field shifts were previously observed in a prototypic peptide of the Kunitz family, Basic Pancreatic Trypsin Inhibitor (BPTI, sequence identity with HCIQ2c1 32%) [32]. The ^1^H signals of H_2_C^β^ (0.9 and 0.4 ppm) and H_2_C^γ^ (0.2 and –0.5 ppm) groups of the Lys10 side chain were also significantly up–field shifted due to interactions with aromatic rings of Tyr23 and Phe34, while the ^1^H^β3^ resonance of Cys56 was shifted to 1.8 ppm due to ring-current effect from the Phe24 side chain (Figure 4C and Figure 4D, respectively).

The measured ^1^H, ^15^N, and ^13^C chemical shifts analyzed by the TALOS software [30], ^3^J_H_^N^_H_^α^ coupling constants, temperature gradients of amide protons, and NOE connectivities provided information about the secondary structure of HCIQ2c1 (Figure 4E). According to these data, the *C*-terminal region forms the long α-helix spanning Thr48–Arg57 residues. The *N*-terminal region Asn4–Ser7, demonstrating the sequence of small (<6 Hz) ^3^J_H_^N^_H_^α^ values, probably adopts the 3_10_-helix conformation, whereas Phe19–Asp25 and Lys30–Tyr36 regions form the β-strands (Figure 4E).

**Figure 5:**
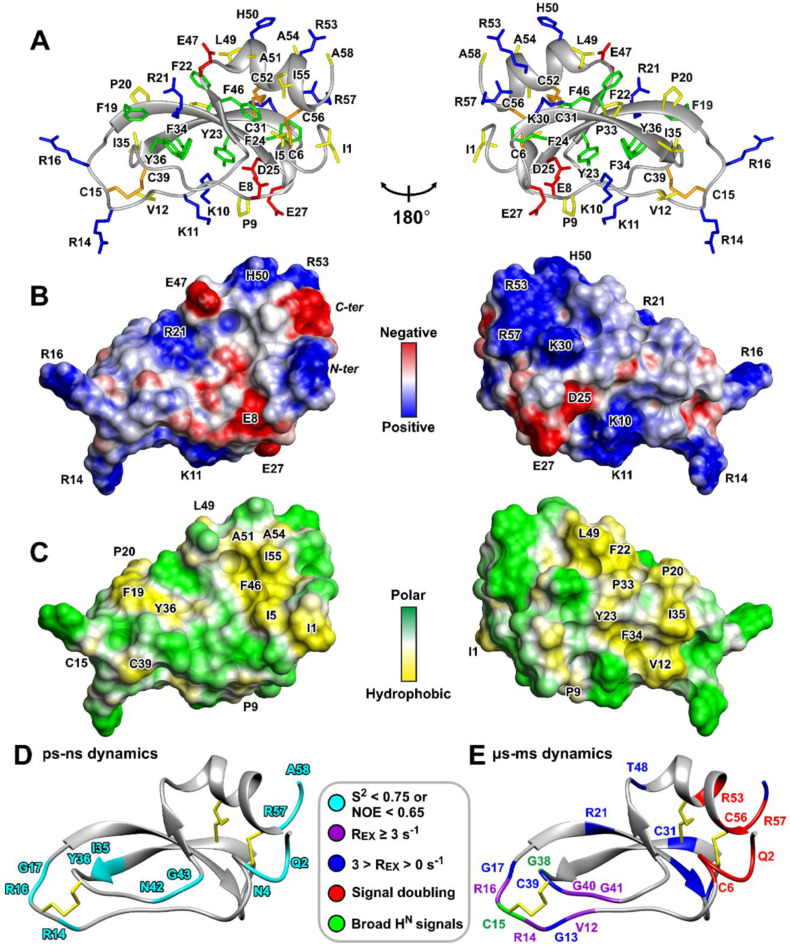
The spatial structure and backbone dynamics of HCIQ2c1 in aqueous solution. (**A**) Two–sided view of the HCIQ2c1 molecule. Positively charged (+His), negatively charged, hydrophobic, and aromatic residues are colored by blue, red, yellow, and green, respectively. The disulfide bonds are shown in orange. (**B**,**C**) Two-sided view of the molecular surface of HCIQ2c1. Electrostatic (**B**) and molecular hydrophobicity [34] (**C**) potentials are shown. (**D**) Regions with high-amplitude mobility on the ps–ns time-scale (where S^2^ < 0.75 or ^15^N–{^1^H} NOE < 0.65) are shown in cyan color. (**E**) Regions with mobility on the μs–ms time-scale are shown in purple (R_EX_ ≥ 3.0 s^−1^ or R_1_ × R_2_ > 20.0 s^−2^) and blue (3 ≥ R_EX_ > 0 s^−1^) colors. The residues demonstrating line-broadening due to intense μs–ms time-scale motions (Cys15 and Gly38) are shown in green color. Regions where signal doubling was observed due to mobility on the millisecond time-scale are shown in red color.

A set of 20 HCIQ2c1 structures (Figure S4 and Figure 5A) was calculated in the CYANA program [33] using the experimental data listed in Table S2. The core of the HCIQ2c1 molecule is represented by a three-stranded antiparallel β-sheet (β1 Phe19–Asp25, β2 Lys30–Tyr36, and β3 Asn45–Phe46) (Figure 4E,F and Figure 5A). The β1 and β2 strands are connected by turn Ser26–Gly29 forming the β-hairpin. The helical elements (Asn4–Ser7 and Thr48–Arg57) are connected with each other and with the β2 strand by Cys6–Cys56 and Cys31–Cys52 disulfides.

The two glycine-rich fragments Val12–Ser18 and Ile35–Gly43 are connected by the third disulfide bond Cys15–Cys39. These fragments lack regular backbone dihedral angles and hydrogen bonds, and demonstrate lower convergence in the calculated set of structures (Figure S4). According to common nomenclature for Kunitz-type protease inhibitors, these irregular fragments are designated as L-loops, L_1_ is the canonical protease binding loop and L_2_ is the weak contact site, respectively. The L_1_ loop contains the protease binding site spanning the P_3_ to P_3_’ positions [35] (Arg14–Phe19) and includes the solvent-exposed reactive P_1_-P_1_’ site (Arg16–Gly17, Figure 4E,F red) that is resistant to proteolytic attack [36]. A positively charged residue (Arg/Lys) at the P_1_ position and a small side chain residue (Gly/Ala) at the P_1_’ position are necessary for inhibition of trypsin and trypsin-like enzymes. The L_1_ and L_2_ loops also contain several hydrophobic residues Phe19, Pro20, Ile35, and Tyr36, which interact with the hydrophobic patch on the protease molecule [37]. The stabilizing disulfide bond Cys15–Cys39 ensures that, during catalysis, the two halves of the hydrolyzed inhibitor peptide chain do not dissociate and can even rejoin [36].

The disulfide bond pattern proposed for the HCIQ2c1 molecule (C1–C6, C2–C4, and C3–C5, Figure 4E,F and Figure S4) was confirmed by the presence of H^α^–H^β^ NOE-contacts in 2D NOESY spectra and is characteristic of Kunitz-type peptides. In addition to the three disulfide bonds, the spatial structure of HCIQ2c1 is stabilized by 21 backbone–backbone hydrogen bonds revealed by Δδ^1^H^N^/ΔT measurements and confirmed during the calculation of preliminary structures. There are also nine backbone-side chain (H^η21^ Arg21–CO Glu47, H^N^ Phe24–O^δ1^ Asn44, H^N^ Glu27–O^δ1^ Asp25, H^N^ Gly41–O^η^ Tyr36, H^δ21^ Asn42–CO Pro9, H^N^ Asn44–O^ε1^ Gln8, H^δ21^ Asn44–CO Gln8, H^δ22^ Asn44–CO Phe24, H^N^ Ala51–O^γ1^ Thr48) and one side chain-side chain (H^η^ Tyr36–O^δ1^ Asn45) hydrogen bond.

The resulting HCIQ2c1 structure is shown in Figure 5A and its molecular surface properties (electrostatic potential and hydrophobicity) are illustrated in Figure 5B,C. The molecule at neutral pH has a theoretical net positive charge of +4 (assuming that His50 side chain is uncharged). Analysis of the HCIQ2c1 surface properties showed that the oppositely charged and hydrophobic residues are segregated. The two groups of the positively charged residues (Lys10, Lys11, Arg14, Arg16 and Lys30, His50, Arg53, Arg57) and negatively charged Asp25 and Glu27 surround the relatively large hydrophobic cluster formed by the side chains of Val12, Phe19, Pro20, Phe22, Tyr23, Pro33, Ile35, Tyr36, and Leu49. The opposite side of the HCIQ2c1 molecule includes the second, more compact hydrophobic cluster formed by the side chains of Ile1, Ile5, Phe46, Ala51, Ala54, and Ile55, which is bounded by the negatively charged groups of Glu8, Glu47, and *C*-terminal Ala58 and by positively charged groups of the *N*-terminal Ile1, Arg21, and His50 side chain. This second hydrophobic cluster is formed around the highly conserved Phe46 residue, which in homologues peptides is required for the interaction with proteases [37]. The Arg14 and Arg16 residues, which are found in many Kunitz-type peptides (see below), contribute to the positive electrostatic potential on the protease-binding loop.

Due to the location of the hydrophobic clusters on the opposite sides of the molecule, the structure of HCIQ2c1 does not exhibit amphipathic properties. Therefore, we do not expect a strong membrane affinity for the HCIQ2c1 molecule, as has been observed for some other peptides targeting TRPA1 [28]. At the same time, one of these hydrophobic clusters may be involved in the peptide interaction with the TRPA1 channel.

### 2.8. Backbone Dynamics of HCIQ2c1

To investigate the conformational plasticity of the HCIQ2c1 molecule, we measured relaxation parameters of the backbone ^15^N nuclei (R_1_ and R_2_ relaxation rates and ^15^N–{^1^H} heteronuclear NOEs) at 60 MHz, pH 4.5, and 30 °C (Figure S5). Analysis of these data using a so-called ’model-free’ approach revealed parameters describing the overall reorientation of the molecule in solution (rotational correlation time, τ_R_), ‘fast’ ps–ns time-scale motions (generalized order parameters, S^2^, Figure 5D and Figure S5) and ‘slow’ μs–ms conformational fluctuations (exchange contributions to the R_2_ relaxation rates of ^15^N nuclei, R_EX_, Figure 5E and Figure S5). Calculations using an isotropic rotational diffusion model yielded a τ_R_ of ~2.9 ns, which corresponds to the reorientation of a globular particle with a hydrodynamic Stokes radius (R_H_) of ~17 Å, in agreement with the dimensions of the HCIQ2c1 molecule (34 × 27 × 23 Å). Thus, the HCIQ2c1 molecule probably exists in solution in a monomeric form.

The low values of S^2^ (<0.75) or ^15^N–{^1^H} NOE (<0.65) revealed regions of the peptide with high amplitude of motions on the ps–ns timescale (Figure 5D). High-amplitude motions were observed in the *N*- and *C*-terminal regions (Gln2–Asn4 and Arg57–Ala58) and for several residues belonged to or located near the L_1_ and L_2_ loops (Arg14–Gly17, Ile35, Tyr36, Asn42, and Gly43). In contrast, the HCIQ2c1 structure in other regions, excluding the L-loops and the *N*- and *C*-termini, (Phe19–Tyr36, Asn45–Cys56) was quite rigid on the ps–ns timescale (S^2^ = 0.85 ± 0.03, NOE = 0.72 ± 0.04, mean ± S.D.).

The presence of R_EX_ contributions and large values of the R_1_ × R_2_ product (>20.0 s^−2^ [38]) revealed regions of ‘slow’ conformational fluctuations on the μs–ms time-scale (Figure 5E). The regions of significant μs–ms motions characterized by large R_EX_ values (≥3 s^−1^) were observed in the L_1_ and L_2_ loops. The L_1_ loop includes residues with the highest R_EX_ values: Val12 (15.5 s^−1^), Arg14 (17.6 s^−1^), and Arg16 (5.8 s^−1^) (Figure 5E and Figure S5). The observed broadening of the ^15^N-HSQC cross-peaks of the Cys15 and Gly38 residues (Figure 4A) probably is originated by the same conformational exchange process. On the other hand, μs-ms fluctuations with smaller intensity (0 < R_EX_ < 3 s^−1^) were observed in the β-hairpin formed by the β1 and β2 strands (residues Arg21–Cys31). In addition, the small R_EX_ value was observed for the spatially close Thr48 residue. Probably these μs-ms conformational fluctuations are consequence of the millisecond time-scale exchange process observed in the *N*- and *C*-terminal peptide fragments and manifested by the signal doubling (Figure 5E, red). It should be noted that the *C*-terminal region is linked to the β-hairpin by Cys31–Cys52 disulfide.

The obtained data showed that the L_1_ and L_2_ loops, linked by the Cys15–Cys39 disulfide exhibit high-amplitude mobility on two time-scales (ps–ns and μs–ms). This mobility likely plays a functional role in the inhibitory activity of HCIQ2c1, as it may allow the P_1_–P_1_′ residues to successfully integrate into the active site of proteases [37]. Another region of HCIQ2c1 involved in motions on the two time-scales (ps–ns and milliseconds) is formed by spatially close *N*- and *C*-terminal fragments also connected by the disulfide bond (Cys6–Cys56). It is likely that ‘slow’ conformational fluctuations propagate from this region to the region of the β-hairpin via the Cys31–Cys52 disulfide. Some of these motions may also be important for the interaction of HCIQ2c1 with the TRPA1 channel.

### 2.9. Computer Modeling of the TRPA1/HCIQ2c1 Complex

As it was shown in Section 2.6 above, HCIQ2c1 allosterically modulates TRPA1, probably by binding to and stabilizing the open channel conformation, preventing transition to the closed and ‘hyperactivated’ states. At the same time, HCIQ2c1 is a relatively large positively charged (+4) peptide, uncapable to easily penetrate the membrane and attack a cytoplasmic or intramembrane TRPA1 sites. Therefore, we hypothesized that HCIQ2c1 binds to the extracellular interface of the channel and simultaneously contacts multiple extracellular loops on the VSLD and/or one or two nearby PD subunits. Exclusive binding of HCIQ2c1 to the PD is unlikely for two reasons. (1) In the case of tight binding, one would expect a hindered ion passage through the channel, which is not the case. (2) The HCIQ2c1 molecule is quite large, so if it contacts one or two PD subunits, it will also contact one of the VSLDs. Exclusive binding of HCIQ2c1 to a single VSLD is also unlikely for the same reason.

To gain a deeper understanding of the structural basis of the interaction between HCIQ2c1 and the open state of the TRPA1 channel, an ensemble protein–protein docking was employed. The obtained solutions were filtered, four most probable distinct binding modes were studied using unbiased MD simulations. Based on the analysis of intermolecular contacts in the obtained trajectories, we selected the most probable binding mode, potentially explaining the mechanism of the HCIQ2c1 action on the TRPA1 channel.

#### 2.9.1. TRPA1–HCIQ2c1 Ensemble Docking

**Figure 6:**
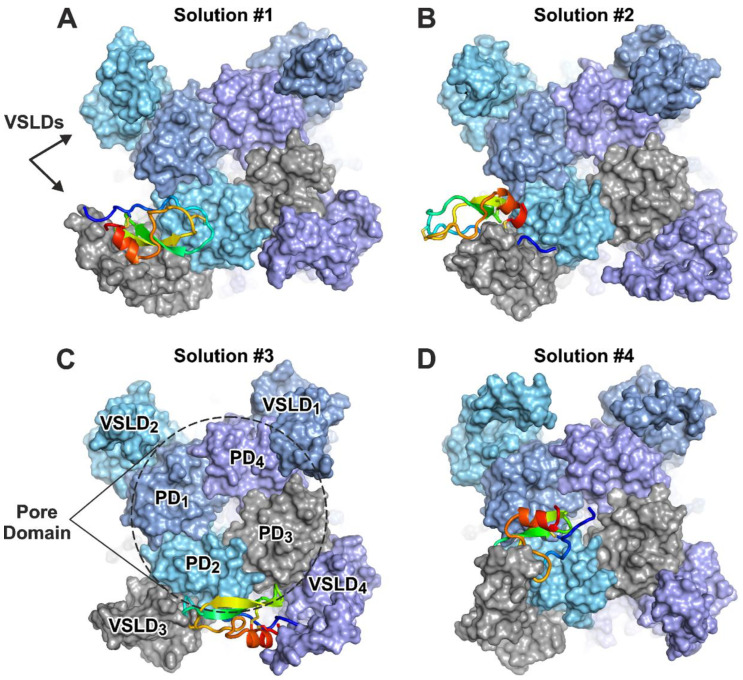
Best docking solutions of the TRPA1/HCIQ2c1 complex, viewed from the extracellular side. The four subunits of the open rat TRPA1 channel are shown by differently colored surfaces (**A**–**D**). Each subunit includes a ¼ of the pore domain (PD; in center) and the voltage-sensing-like domain (VSLD; distal). The HCIQ2c1 backbone is spectrum colored from blue (*N*-terminus) to red (*C*-terminus). Disulfide bonds are shown in yellow. The glycans on the VSLDs were modeled in MD but omitted in docking.

The ensemble docking approach starts with a series of conformations of both protein partners to account for their molecular flexibility:

1. Conformational clustering of the HCIQ2c1 NMR ensemble yielded four diverse structures, which underwent MD simulations in aqueous solution with 0.15 M NaCl for 500 ns each. The four trajectories were combined into an aggregate 2000 ns trajectory. Repeated clustering resulted in 49 peptide conformations used for docking.
2. The open state of the rat TRPA1 channel was modeled based on the previously resolved cryo-electron microscopy structure of the human orthologue (PDB code 6V9X [6]). A 500 ns MD simulation in an explicit 1-palmitoyl-2-oleoyl-phosphatidylcholine (POPC) lipid bilayer (aqueous solution, 0.15 M NaCl) was performed. Clustering of this trajectory by conformations of extracellular residues yielded 131 TRPA1 conformations. Glycans were removed from TRPA1 before clustering, and glycan-free structures were used for subsequent docking.
3. Ensemble protein–protein docking was initiated as a series of 131 × 49 = 6419 independent docking runs, each yielding the top 100 solutions, resulting in a total ensemble of 641,900 probable structures of the TRPA1/HCIQ2c1 complex.
4. This vast ensemble was filtered according to certain criteria (Table S3), including the area of the interaction interface (*S*), the complementarity of hydrophobic/hydrophilic properties at the interface (*Cp*) [39], and the total number of specific intermolecular contacts (interactions) that stabilize the complex, including ionic (at least six), H-bonds, stacking, and π–cation interactions. Based on these criteria, just four dissimilar docking solutions were selected (Figure 6 and Figure S6) for further assessment.

In agreement with our expectations, in each of these solutions, HCIQ2c1 contacted multiple channel’s domains/subunits simultaneously. In solutions ## 1, 2, and 4, the peptide interacted with two neighboring pore domain subunits (PD_1_ and PD_2_) and with the nearby VSLD_3_ (numbered counterclockwise when viewed from the extracellular side, Figure 6A,B,D). At the same time, the peptide orientation was quite different, such that the *N*-terminal region and the *C*-terminal helix contacted the VSLD_3_ (solution #1), PD_2_ (solution #2), or PD_1_ (solution #4). In contrast, in solution #3, HCIQ2c1 bound two VSLDs of subunits 3 and 4 and two PDs of subunits 2 and 3 (Figure 6C).

#### 2.9.2. Molecular Dynamics of the TRPA1/HCIQ2c1 Complexes Points the Most Probable Binding Mode

MD simulations were performed for each of the four selected solutions to assess their overall stability, behavior, and evolution of the binding mode during the MD. Simulations were conducted in the POPC lipid membrane in the presence of glycans on TRPA1 (two per VSLD). To increase conformational sampling, MD systems ## 1 and 2 included four HCIQ2c1 molecules per channel, one per VSLD. In solution #3, the peptide contacted two VSLDs, so MD was set up with two HCIQ2c1 molecules per channel. In solution #4, the *N*-terminus of the peptide interacted with the channel pore, so we ran two independent MD replicas with one HCIQ2c1 molecule per channel in each (trajectories ## 4 and 5). The replicas were created by random velocity assignment after energy minimization.

MD (500 ns each) was simulated for each configuration (Table S4 and Figure S7), and 250–500 ns segments were kept for analysis (Table S5). The root-mean square deviation (RMSD) calculated relative to the starting position showed that not all HCIQ2c1 instances retained the initial binding mode. We considered a trajectory stable if the average RMSD value over the 250–500 ns time window was ≤1.0 nm. Using this cutoff, only 50% (2 out of 4) of the peptide instances were stable for solutions ## 1 and 2. Solution #3 yielded no stable complexes at all, while all two replicas for solution #4 were stable. Interestingly, for solution #2, two peptide molecules 2–2 and 2–3 (# of MD trajectory—# of peptide molecule) drifted from their initial positions and aggregated at the top of the PD, contacting the third stable peptide molecule 2–1.

**Figure 7:**
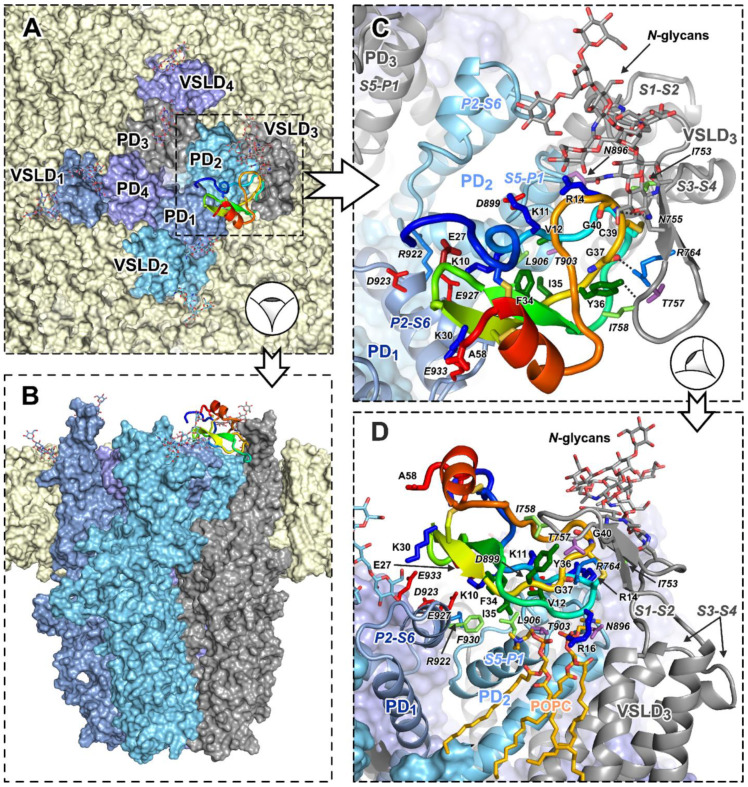
MD snapshot of the best TRPA1/HCIQ2c1 complex (5–1, see Table S5). Colors and designations are the same as in Figure 6. The *N*-glycan groups attached to Asn749 and Asn755 on the S1–S2 loop of each VSLD are represented as sticks and colored by atom type. (**A**,**B**) Top and side view on the simulation system. Membrane lipids are shown as a surface; water and ions are omitted. In (**B**), the nearby membrane slab is hidden for clarity. (**C**,**D**) Close-up top and side views of the TRPA1/HCIQ2c1 complex. Active residues are shown as sticks and colored according to the residue type: positively charged—blue, negatively charged—red, polar—violet, hydrophobic/aromatic—green, cysteines—yellow. Channel residues are italicized and shown in thinner and lighter sticks. POPC lipids and glycine residues are shown with sticks colored by atom type.

Analysis of the contacts and interaction areas of the peptide with the channel and lipid membrane identified three TRPA1/HCIQ2c1 stable complexes 1–4, 2–1, and 5–1 (Table S5, bold, Table 1) shown in Figure 7 and Figure S8. In all three complexes, HCIQ2c1 retains three-point channel interaction, seeded in the initial docking solutions: S1–S2 of the VSLD_3_, S5–P1 (and P2–S6 for complexes 1–4 and 2–1) of the PD_2_, and P2–S6 loop of the neighboring pore subunit PD_1_. In complexes 1–4 and 2–1, this interaction was relatively short-lived (up to ~30% of the total MD length), whereas in complex 5–1, the peptide interacted with the PD_1_ for up to 75% of the MD time (Table 1). Stable HCIQ2c1 interactions with another extracellular loop of the VSLD_3_, S3–S4, were observed only in complex 2–1. Interestingly, long-lived peptide–lipid interactions (salt bridges between positively-charged peptide side chains and POPC phosphate) were observed in complexes 2–1 and 5–1 (Table 1). In complex 2–1, the Lys30 side chain formed such bond throughout 40% of the MD trajectory, whereas in complex 5–1, the Arg16 side chain simultaneously formed two or three ionic bonds with different lipid molecules throughout almost entire MD (Figure 7D).

Based on the number and lifetime of the observed HCIQ2c1 intermolecular contacts (Table 1), the most probable complex (5–1) was identified (see Figure 7). Unlike 1–4 and 2–1, in 5–1 complex HCIQ2c1 does not extensively contact *N*-glycans at Asn749 and Asn755 residues (VSLD S1–S2 loop). These *N*-glycans are very flexible, so 5–1 complex stability seemingly does not depend on these weak peptide–glycan interactions, but rather on more stable peptide–protein and peptide–lipid interactions. There are several long-lived ionic bonds (Asp899(S5–P1_2_)–Lys11, Arg922(P2_1_)–Glu27, Glu927(P2_1_)–Lys10, Glu933(P2_1_)–Lys30), and π-cation interaction Arg764(S1–S2_3_)–Tyr36. In addition, complex 5–1 is stabilized by several H-bonds and hydrophobic contacts with the large hydrophobic cluster on the HCIQ2c1 surface that belts the L_1_ and L_2_ loops (residues Val12, Cys15, Phe34, Ile35, Tyr36, and Cys39).

The region of the L-loops of the HCIQ2c1 molecule became sandwiched between the S5–P1 loop of the PD_2_ from below and the long S1–S2 loop of the VSLD_3_ from above (Figure 7C,D). This significantly limits the mobility of the peptide and prevents it dissociation from the binding site. Intermolecular interactions observed in complex 5–1 likely contribute to the functional modulation of TRPA1 by HCIQ2c1 and provide a structural basis for understanding the mechanism of the peptide’s action. We suggest that it is the simultaneous interaction with the PD and VSLD that provides the ‘normalizing’ action of HCIQ2c1 on the open state of TRPA1.

## 3. Discussion

**Figure 8:**
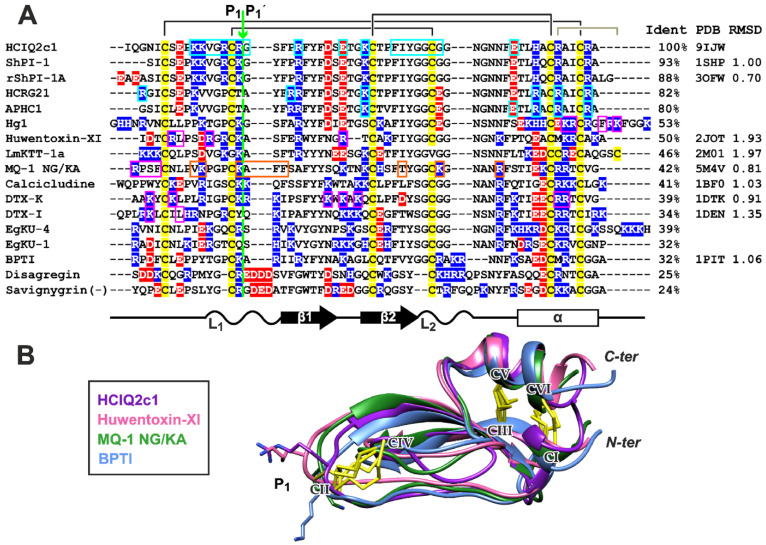
Comparison of HCIQ2c1 with other Kunitz-type peptides. (**A**) Multiple sequence alignment. Positively and negatively charged residues are indicated by blue and red squares respectively; cysteines are shown in yellow. The green arrow shows a site resistant to proteolytic cleavage, but not all listed peptides demonstrate protease inhibition activity. Cyan boxes indicate the residues involved in TRPV1 inhibition by HCRG21 and APHC1, and regions responsible for the HCIQ2c1 binding to rat TRPA1 in complex 5-1. Magenta boxes indicate the residues involved in interaction with K^+^-channels. Orange boxes show the residues critical for mambaquaretin-1 (MQ-1) interaction with the type-2 vasopressin receptor. Conserved disulfide bonds and secondary structure elements defining Kunitz-fold are shown. Black arrows and white box indicate the β-strands and α-helix, respectively; wavy lines show the L_1_ and L_2_ loops. Sequence identity with HCIQ2c1 (%), PDB codes, and root mean square deviation (RMSD) values calculated over C_α_-atoms in regions of conserved secondary structure (19–36, 45–57) are shown on the right. (**B**) Comparison of the spatial structures of HCIQ2c1 with other Kunitz-type peptides (see legend for color code). The backbones of the peptides are shown as ribbons, cysteines are shown in yellow, and conserved Arg/Lys residues at position P_1_ of the protease binding site are shown as sticks.

Kunitz-peptides possess one of the most evolutionarily ancient and conserved structural motifs widespread in venomous terrestrial and marine organisms [40]. These peptides are one of the most represented groups of sea anemone toxins. They are encoded by multigene families and form a great diversity of peptide isoforms in the sea anemone venom [21,41,42]. Among sea anemone Kunitz-type peptides, the spatial structure previously was studied only for ShPI-1 from *Stichodactyla helianthus*, which inhibits trypsin and trypsin-like proteases [43]. The main feature of the Kunitz-domain is three conserved intradomain disulfide bonds (CysI–CysVI, CysII–CysIV, CysIII–CysV) which connect the conserved β-hairpin (β_1_–β_2_ strands) and elongated *C*-terminal α-helix (Figure 8A). This structure forms two loops (L_1_ and L_2_) connected by the CysII–CysIV disulfide, which often constitutes the protease-binding site.

Here, we report the spatial structure of the second Kunitz-type peptide from sea anemone, HCIQ2c1 from *Heteractis magnifica*. The determined HCIQ2c1 structure can be relatively well superimposed with Kunitz-type peptides from various organisms by regions of conserved secondary structure (Figure 8, sequence identity 32–93%, average RMSD ~ 1.2 Å). This structural conservation leads to a good overlap of the secondary structure elements and results in a coincidence of the position of the CysII–CysIV disulfide bond and the key dipeptide P1–P1’ (Arg16–Gly17 in HCIQ2c1) responsible for the protease inhibition (Figure 8B).

HCIQ2c1 is highly efficient in inhibiting serine proteases (trypsin *K*_i_ ~50 nM [21]) and demonstrates allosteric modulation of the TRPA1 channel (Figure 2 and Figure 3). The combination of such activities is not surprising, as sea anemone Kunitz-peptides often combine the inhibition of proteases with action on ion channels. For example, the recombinant analogue of ShPI-1 (rShPI-1A, Figure 8A) inhibits the potassium voltage-gated channels K_V_1.1, K_V_1.2, and K_V_1.6 [44], APEKTx1 from *Anthopleura elegantissima* selectively interacts with K_V_1.1 [45], kalicludine peptides from *Anemonia sulcata* compete with snake DTX-I (see below) for the binding to the K_V_1.2 channel [46]. APHC1-3 [47,48] and HCRG21 [49,50] peptides from *H. magnifica*, closely related to HCIQ2c1, inhibit the TRPV1 channel. The ring of positively charged residues of APHC1, Lys 28, Arg48, Arg51, and Arg55 (as well as Arg1 and Arg18 for HCRG21, Figure 8A, cyan boxes) [48,50] is thought to play a crucial role in the binding. Notably, HCRG21 does not act on the K_V_1.1–1.6, Shaker-IR and hERG channels, while other HCIQ2c1 homologues, HCRG1 and HCRG2 from the same anemone [51] block the K_V_1.3 channel [52]. HCIQ4c7, another *H. magnifica* peptide related to HCIQ2c1, inhibits the P2X7R ion channel [22].

Among the scorpion Kunitz-type toxins with inhibitory activity against proteases, the Hg1 peptide from *Hadrurus gertschi* selectively blocks the K_V_1.3 channel (weak inhibitory activity at the calcium-activated K_Ca_2.3 potassium channel was also described [53]). It was proposed that the *C*-terminal region of the toxin is responsible for interaction with K^+^ channels (Figure 8A, magenta boxes). The toxin LmKTT-1a from the Chinese swimming scorpion *Lychas mucronatus* has an atypical disulfide bond pattern, but retains the Kunitz–fold and blocks the K_V_1.3 channel [53]. Kunitz-type toxin Huwentoxin-XI from the Chinese bird spider *Haplopelma schmidti* simultaneously inhibits trypsin (K_i_ ~ 70 nM [54]) and the K_V_1.1–3 channels. In contrast to Hg1, the active site of Huwentoxin-XI includes the *N*-terminal positively charged residues [55] (Figure 8A).

Several Kunitz-type toxins from green and black mambas are active at ion channels and membrane receptors but lack protease inhibitory activity. Mambaquaretin-1 (MQ-1) from the Eastern green mamba *Naja angusticeps* shows weak inhibition of the K_V_1.1 channel, for which the first four residues have been shown to be essential (Figure 8A), and selectively inhibits the type-2 vasopressin receptor (V2R) [56]. In this case, the active residues are located in the middle of the molecule (Figure 8A, orange boxes). Interestingly, substitution of residues in the P1–P1’ position (Asn–Gly for Lys–Ala) significantly increases the toxin activity towards trypsin but decreases the affinity for V2R. Calcicludine from Eastern green mamba interacts with the outer vestibule of Ca_V_1.1–3 channels and stabilizes them in a nonconducting state via allosteric interactions [57]. Dendrotoxins DTX-I and DTX-K from *Dendroaspis polylepis* (black mamba) interact with the K_V_1.1 channel through a set of positively charged residues located at the *N*-terminus and at the top of the β-hairpin [58]. Dendrotoxin α-DTX from *Dendroaspis angusticeps* shows the weak inhibitory activity against acid-sensing ion channels (ASICs) [59].

Two peptides EgKU-1 and EgKU-4 from the parasitic cestode *Echinococcus granulosus* block K_V_ channels in dorsal root ganglion (DRG) neurons and ASICs [60]. Remarkably, only EgKU-4, which has Arg–Ser at the P1–P1’ position, can inhibit trypsin with K_i_ ~ 50 nM. Disagregin from soft tick *Ornithodoros moubata* specifically binds to the αIIb-β3 integrin found on platelets and inhibits the platelet aggregation [61]. The sequence of the homologous peptide savignygrin from *Ornithodoros savignyi* has the classical integrin recognition fragment (Arg-Gly-Asp) and also blocks the platelet aggregation [62].

According to the electrophysiological data (Figure 2 and Figure 3), HCIQ2c1 allosterically modulates TRPA1 and ‘normalizes’ its function. The binding of the peptide to the open channel prevents TRPA1 transition to the closed and ‘hyperactivated’ states or impedes the dissociation and association of non-covalent and covalent agonists. A model of the TRPA1/HCIQ2c1 complex (Figure 7) agrees well with this peptide’s activity. The simultaneous interaction of HCIQ2c1 with the VSLD and two PD fragments from the different TRPA1 subunits, as well as with the membrane lipids likely imposing restraints to the possible orientation of the TRPA1 domains in the membrane and packing of the PD. This may shift the conformational equilibrium toward a particular channel state (e.g., open state). Indeed, a comparison of the open and closed TRPA1 structures reveals that the major movements occurring during the channel activation are the rearrangement of the PD packing including the changes in its extracellular interface and the rigid body rotation of the VSLDs around the PD [6].

The non-covalent agonists, like GNE551, interact with the TM region of the TRPA1 channel by hijacking the pocket located between the VSLD and PD, between helices S4 and S5, above the S4–S5 linker [7]. In the closed TRPA1 channel, this pocket is occupied by the phospholipid molecule [7]. At the same time, one of the binding sites for small-molecule TRPA1 antagonists (e.g., HC-030031 [8] and tetrahydrofuran-based antagonists [9]) is located directly under this pocket, below the S4–S5 linker. At this point, we do not know exactly what changes in the TRPA1 structure correspond to the observed ‘hyperactivated’ state, but the decreased sensitivity to the HC-030031 antagonist suggests that the changes may occur in the binding sites located between the VSLD and PD. In this case, the HCIQ2c1 binding to the extracellular interfaces of both VSLD and PD could affect the agonists and antagonists binding sites located deeper in the membrane between the domains.

The distribution of TRPA1-interacting residues in the HCIQ2c1 sequence is similar to the distribution of MQ-1 residues required for binding to V2R (Figure 8A, cyan and orange boxes). At the same time, the model proposed for the TRPA1/HCIQ2c1 complex resembles the previously proposed TRPV1/APHC1 model [48]. In both models, the peptides interact at the interface of two PD fragments from different subunits, linking them together. However, the orientation of the peptides is significantly different. In the case of APHC1, the main binding site is the *C*-terminal α-helix, while the HCIQ2c1 molecule has the opposite orientation and interacts with the cleft on the PD surface by the β-hairpin (Figure 7).

Interestingly, the two suggested HCIQ2c1 sites responsible for the interaction with TRPA1 (the region of L_1_ and L_2_ loops that interacts with the VSLD_3_, and the *N*- and *C*-terminal regions that interact with the PD_1_) coincide with two hot-spots of dynamics. According to the ^15^N relaxation data (Figure 5D,E), these regions of the peptide exhibit the motions on two timescales (ps–ns and μs–ms). So, these motions may be functionally important for the interaction with TRPA1 through adaptation of the peptide structure to the extended binding site consisting of two mutually mobile parts (VSLD_3_ and PD_1_).

According to the proposed model, the long extracellular S1–S2 loop of VSLD is involved in the interaction with HCIQ2c1. This loop represents a binding site for another TRPA1 ligand, the spider cystine knot toxin ProTx-I [10]. The S1–S2 loop contains two N-glycans attached to Asn749 and Asn755 (numbering is given for the rat channel). Moreover, the Asn749Gln mutation resulting in deletion of one of the N-glycans changes the sensitivity of the channel to covalent and non-covalent agonists [63]. These data also support the proposed mechanism of the HCIQ2c1 action on TRPA1.

AITC is a natural TRPA1-agonist, which induces spontaneous pain behavior, thermal and mechanical hyperalgesia of mice through activation of TRPA1-positive nociceptors [26]. Moreover, the application of TRPA1 agonists to the skin leads to the development of local edema and activates local immune cells to amplify neurogenic inflammation [64]. HCIQ2c1 slightly suppressed sensitivity of mice to temperature increase in the Hot plate test (Figure 1A) and inhibited AITC-induced pain reactions and paw swelling (Figure 1B,C). We suggest that both modes of the HCIQ2c1 activity observed on the TRPA1 channel by electrophysiology may translate into analgesic and anti-inflammatory activity in vivo. On the one hand, the suppression of the ‘hyperactivation’ state of TRPA1 characterized by reduced sensitivity to antagonists (Figure 3) can directly reduce TRPA1 nociceptive responses. On the other hand, the prolonged (increased) channel activity observed in the experiments with diclofenac (Figure 2) may lead to desensitization of the TRPA1-containing neurons and could also cause suppression of pain signaling. Indeed, two peptides Ms-9a from sea anemone *Metridium senile* and Ueq 12-1 from *Urticina eques* significantly potentiate the TRPA1 response to various agonists and simultaneously possess analgesic activity in vivo [19,20,65]. Moreover, another TRPA1 ligand showing very potent analgesic effect, Phα1β toxin from spider *Phoneutria nigriventer*, has recently been shown to potentiate the TPRA1 responses and block the receptor desensitization, similar to that observed for HCIQ2c1 [28]. Of note, both TRPA1 ligands used (AITC and diclofenac) are exogenous, and at present we cannot predict what mode of HCIQ2c1 action will be active upon the TRPA1 stimulation by endogenous inflammatory mediators.

HCIQ2c1 also reduced the mice sensitivity to the irritant action of the TRPV1 agonist capsaicin (Figure 1D), but did not influence the TRPV1 channel activity [22]. Given the possible co-localization of TRPA1 with TRPV1 and the mutual influence of the channels in the membranes of sensory neurons [66], we can explain the observed analgesic effect.

## 4. Conclusions

We produced a recombinant analog of HCIQ2c1, the Kunitz-type peptide from *Heteractis magnifica*. 3D structure and dynamics of HCIQ2c1 were determined by NMR spectroscopy. The peptide prolongs the TRPA1 response and prevents the channel transition to an inhibitor insensitive ‘hyperactivated’ state. HCIQ2c1 exhibits analgesic activity in the Hot plate test, decreases AITC- and capsaicin-induced pain, and suppresses AITC-induced inflammation in vivo. In the modelled TRPA1/HCIQ2c1 complex, the peptide interacts with the outer loops of the VSLD and PD, stabilizing the channel in an open conformation and possibly influencing the agonist and antagonist binding.

To the best of our knowledge, HCIQ2c1 is the second sea anemone Kunitz-type peptide with determined 3D structure and the first Kunitz-type ligand of the TRPA1 channel. The HCIQ2c1 peptide may be an effective analgesic and anti-inflammatory agent or used as a tool to study the TRPA1 channel.

## 5. Materials and Methods

### 5.1. Bacterial Expression of HCIQ2c1

Recombinant HCIQ2c1 was obtained as described in [21] using BL21 (DE3) strain of *Escherichia coli* and *pET32b/HCIQ2c1* plasmid. In order to produce the ^15^N-labeled peptide, transformed cells were cultured on Luria Bertani medium to an optical density (OD_600_) of 0.6. Cells were centrifuged at 5000× *g* for 5 min and the cell pellet was resuspended in 300 mL of M9 minimal medium (6 g of Na_2_HPO_4_, 3 g of KH_2_PO_4_, 0.5 g of NaCl, 2 g of ^15^N NH_4_Cl (Merck KGaA, Darmstadt, Germany), 240 mg of anhydrous MgSO_4_, 11 mg of CaCl_2_, 2 g of Glycerol, 2 mg of yeast extract, and 200 μL of 5% thiamine chloride per 1 L of medium, pH 7.4, 100 μg/mL carbenicillin) to a final OD_600_ of 1.2. The expression was induced by an addition of 0.2 mM isopropyl β-D-1-thiogalactopyranoside (IPTG) and cells were cultivated at 13 °C during 48 h. The biomass was harvested via centrifugation at 10,000× *g*, 10 min, 4 °C, resuspended in 30 mL of starting buffer (400 mM NaCl, 20 mM Tris-HCl buffer, pH 8.0) and ultrasonicated on ice. Fusion protein TRX-HCIQ2c1 was purified on a Ni-nitrilotriacetic acid (NTA) agarose (Qiagen, Venlo, The Netherlands) according to the manufacturer’s instructions and cleaved using CNBr overnight at room temperature with a CNBr/protein molar ratio of 600:1 [67]. The recombinant peptide was purified from a reaction mixture on a Jupiter C4 (250 × 10 mm) reverse–phase column (Phenomenex, Torrance, CA, USA) using a linear gradient of acetonitrile (from 0% to 70%) with 0.1% trifluoroacetic acid (TFA) over 70 min with a constant flow rate of 2 mL/min. Fraction containing the peptide was lyophilized.

### 5.2. MALDI TOF/MS Analysis

MALDI TOF/MS spectra of HCIQ2c1 and ^15^N-HCIQ2c1 peptides were recorded using the Ultra Flex III MALDI TOF/TOF mass spectrometer (Bruker, Bremen, Germany) with a nitrogen laser Smart-Beam (355 nm), reflector, and potential LIFT tandem modes of operation. Sinapinic acid was used as the matrix. External calibration was employed using a peptide InhVJ with measured ratio of its mass-to-charge (*m*/*z*) 6107 [68] and its double-charged variant at *m*/*z* 3053.

### 5.3. Animal Studies

The animal studies were performed under the European Commission’s legislation (Directives 86/609/EEC, 2010/63/EU), the National Standard of the Russian Federation “Good Laboratory Practice” (GOST P 53434-2009, Moscow, Russia), and Committee on Ethics of Laboratory Animal Handling No. 05/21, 20 September 2021 protocol (PIBOC FEB RAS). Adult female CD-1 mice weighing 25 ± 2 g were housed under a 12 h light–dark cycle at room temperature and with ad libitum access to food and water. There were seven individuals in each group. HCIQ2c1 at doses of 0.01, 0.1, or 1 mg/kg was administered intramuscularly in the quadriceps muscle of the left hind paw 60 min before each test. Control animals received an equivalent volume (50 µL) of sterile 0.9% saline.

All in vivo data were obtained from the groups containing seven mice (n = 7). Obtained values are expressed as means ± standard error of the mean (S.E.M.). The statistical significance of changes in all groups relative to the control was evaluated using the one-way analysis of variance (ANOVA) followed by a Dunnett’s test using *p* < 0.05 as the level of significance. SigmaPlot 14.0 (Systat Software Inc., San Jose, CA, USA) was applied to determine statistical significance.

#### 5.3.1. Open Field Test

The motor and orienting-exploratory activity of animals was assessed in the Open Field facility (OpenScience, Krasnogorsk, Russia). Arena with a diameter of 63 cm had 12 holes with a diameter of 1 cm to explore the mink activity of rodents. Animals were placed into the center of the illuminated arena and 3 min video recording of the behavior and movement of the animal was immediately started. Analysis of animal motor and orienting-exploratory activity was carried out using the “Minotaur” software (LLC “Neurobiotics”, Zelenograd, Russia). The following parameters were estimated: time of activity, time of immobility, time spent in the central and border zones, average travel speed, traveled distance, vertical activity (rearing), the number of peeps into minks.

#### 5.3.2. Hot Plate Test

Thermal analgesia was tested with a Hot-Plate Analgesia Meter (IITC Life Science Inc., Woodland Hills, CA, USA) set at 52 °C. The animals were placed individually on the preheated hot-plate surface and exposed to heat until nociceptive reaction was registered. Pain threshold was detected as the latency to withdraw or lick the fore or hind paws. The maximum residence time of the animal on the plate did not exceed 60 s.

#### 5.3.3. Capsaicin Test

Capsaicin stock solution was made by dissolving the powder (Sigma-Aldrich, St. Louis, MO, USA) in 96% ethanol. The working capsaicin solution was prepared by mixing the capsaicin stock solution with 0.9% saline at 1:9 ratio. To induce the pain behavior, 20 µL of capsaicin solution (6 µg/mouse) was injected into the plantar skin of the hind paw pad (subplantarly), and then the mouse was put back in the chamber immediately, every animal was observed individually for 15 min. The licking, tucking, scratching, flicking, or biting the injected hind paw were considered as pain-related responses or nociceptive behavior. The pain threshold was detected as the latency to pain-related response. The total amount of time animals spent licking or tucking the injected paw and the number of licking the injected paw were measured.

#### 5.3.4. Allylisothiocyanate-Induced Pain and Paw Edema Test

To induce the pain behavior and paw edema, clean AITC (Sigma-Aldrich, St. Louis, MO, USA) was dissolved in 0.9% sterile saline and then 20 µL of 0.05% AITC solution was injected subplantarly. The mouse was put on the chamber immediately after injection of AITC, and the animal was observed individually for 5 min. The same parameters as for capsaicin test was detected as pain-related response or nociceptive behavior.

To estimate HCIQ2c1 anti-inflammatory activity, the volume of injected paw was measured before and 2, 4, and 24 h after AITC injection. The anti-inflammatory activity of HCIQ2c1 was detected as a decrease of paw volume and Volume Growth Index (%) throughout the entire observation period. Volume Growth Index (%) was calculated using the following formula:

Volume Growth Index (%) = [(V_i_ − V_c_)/V_c_] × 100, (1)

where V_i_ is the volume of the paw after the injection of AITC, V_c_ is the volume of the paw before the injection of AITC.

### 5.4. Electrophysiological Recordings

Preparation of *X. laevis* oocytes, expression of rat TRPA1 receptor, and two-electrode voltage clamp recording with HCIQ2c1 were done as described previously in [28]. The rat TRPA1 receptor [20] was expressed in *X. laevis* oocytes, according to standard procedure. The preparation of the *Xenopus* oocytes at defolliculated stages V–VI was carried out as previously described [69]. The mRNA transcript encoding TRPA1 was synthesized by the mMessage mMachine T7 kit (Cat# AM1344, Thermo Fisher Scientific), according to the protocol for capped transcripts supplied by the manufacturer. Defolliculated oocytes were injected with 20 ng of mRNA and kept for 3–7 days at 18 °C in modified Barth’s solution, supplemented with gentamicin (Cat# G1264, Merck, Darmstadt, Germany) (50 μg/mL) and containing (in mM) 88 NaCl, 1 KCl, 2.4 NaHCO_3_, 0.82 MgSO_4_, 0.33 Ca(NO_3_)_2_, 0.41 CaCl_2_, and 5 HEPES, at pH of 7.4.

Two-electrode voltage-clamp recordings were performed using the TEC-03X amplifier (NPI Electronics GmbH, Tamm, Germany). Prolonged clamping at the positive potentials (Vm) was found to increase the oocyte leakage current and decrease viability, so the holding and inter-pulse (sweep) Vm was set close to the mean oocyte resting potential (−20 mV). The recording of inward/outward currents were made at repeated steps from −20 to −80 mV for 100 ms, following a voltage ramp from −80 mV to +80 mV for 200 ms, and final sweeping to −20 mV for 100 ms. The current amplitudes were measured at the beginning and end of the voltage ramp [20]. The ramp sequence and measurements were repeated every 4 s.

Glass microelectrodes were pulled to ~1 MOhm resistance and filled with 3 M KCl. The output currents and voltage signal were filtered at 50 Hz and digitized at 1 kHz by National Instruments USB-6251 card. The voltage ramps were triggered by an analog output from the same card and the ramp signals were generated by the KMoon FY6900 waveform generator connected to the command input of the amplifier. The data recording and perfusion system control were carried out via WinWCP 5.2.7 (Strathclyde Electrophysiology Software, Glasgow, UK). During the recoding, the oocytes were perfused at 2 mL/min with an ND-96 solution w/o Ca^2+^, containing (in mM) 96 NaCl, 2 KCl, 1 MgCl_2_, and 10 HEPES, at a pH of 7.4. The recordings were performed at room temperature (21–22 °C).

The currents were elicited by an exchange of the solution in the recording bath to an ND-96 solution supplemented with 1 mM diclofenac (Hemofarm a.d., Vršac, Serbia) [70] or 100 µM AITC (Sigma-Aldrich, St. Louis, MO, USA) [71]. The simulation with diclofenac was repeated with 5 min intervals. Several stimulations with AITC were performed in sequential cascades, one cascade per cell. In some experiments, the oocytes were preincubated for 30 s in a 10 µM solution of recombinant HCIQ2c1, and the current was stimulated by the HCIQ2c1+diclofenac/AITC solution. If needed, the solution in the recording bath was exchanged to an ND-96 solution with 10 µM of recombinant HCIQ2c1 or 50 µM of TRPA1 inhibitor HC030031 (Sigma). For diclofenac stimulations, recordings were normalized to control experiments using vehicle solution instead of HCIQ2c1 for the same cell. For AITC stimulations, the recording between cells were normalized by the response to the first application of 100 µM AITC, the amplitude of which was taken as 1.0. The HCIQ2c1 and diclofenac solutions were prepared before each oocyte recording from a 300 µM stock of HCIQ2c1 and 50 mM stock of diclofenac by diluting with Ca^2+^-free ND-96.

The recorded data were processed in Clampfit 10.7 (Molecular Devices, San Jose, CA, USA) and analyzed in GraphPad Prism 9.5.1 (GraphPad Software, San Diego, CA, USA).

### 5.5. NMR Experiments and Spatial Structure Calculation

NMR experiments were performed using the samples containing 0.3 mM of non-labeled or 0.08 mM ^15^N-labeled HCIQ2c1 in 5% D_2_O at pH 4.5. The NMR spectra were acquired on the Bruker Avance-III 600 and 800 spectrometers equipped with a cryoprobes and the Bruker Avance 700 spectrometer equipped with triple-resonance (^1^H,^13^C,^15^N) room-temperature probe at 30 °C. The resonance assignment was performed using standard approach on the basis of 2D ^15^N-HSQC, 3D ^15^N-filtered TOCSY-HSQC (τ_m_ = 80 ms), and NOESY–HSQC (τ_m_ = 100 ms) spectra measured for the ^15^N-labeled peptide, and 2D NOESY (τ_m_ = 120 ms) and 2D TOCSY (τ_m_ = 60 ms) spectra measured for unlabeled sample. Partial ^13^C assignment was performed using 2D ^13^C-HSQC spectrum measured at natural isotope abundance. ^3^J_H_^N^_H_^α^ and ^3^J_H_^β^_N_ scalar coupling constants were measured using 3D HNHA and HNHB spectra, respectively. Additionally, ^3^J_H_^N^_H_^α^ and ^3^J_H_^β^_H_^α^ scalar couplings were estimated via line shape analysis in 2D TOCSY spectrum. 3D TOCSY-HSQC, HNHA, and HNHB spectra were acquired using non–uniform sampling method with 30% of sparse sampling and processed with MDDNMR [72]. Temperature gradients of amide protons (∆δ ^1^H^N^/∆T) were measured from a series of 2D ^15^N-HSQC spectra acquired in the 15–45 °C temperature range.

The secondary structure of HCIQ2c1 was calculated from ^1^H, ^13^C, and ^15^N chemical shifts using the TALOS-N software [30]. The spatial structure was calculated using the CYANA 3.98 program [33]. Upper interproton distance constraints were derived from cross-peaks observed in 2D NOESY (t_m_ = 120 ms) and 3D NOESY (τ_m_ = 100 ms) spectra via a “1/r^6^” calibration. The φ and χ^1^ dihedral angles restraints were obtained from J-couplings and NOE intensities. The hydrogen bonding restraints were applied on basis of temperature coefficients of amide protons, assuming that an amide proton with |Δδ^1^H^N^/ΔT| < 4.5 ppb/°K can participate in the hydrogen bonding. Standard distance restraints were applied to restrain disulfide connectivities.

Relaxation parameters of ^15^N nuclei (longitudinal (R_1_) and transverse (R_2_) relaxation rates and steady–state heteronuclear ^15^N-{^1^H} NOEs) were measured at 60 MHz, pH 4.5, 30 °C using the standard set of ^15^N-HSQC based pseudo 3D experiments. Relaxation data were analyzed using FastModelFree [73]. Isotropic rotational diffusion model was used.

### 5.6. Computer Modeling

#### 5.6.1. Construction of the TRPA1 Channel Model

The open state of the rat TRPA1 channel was homology modelled using the sequence from the UniProt database (protein code: Q6RI86) and the human TRPA1 structure (PDB code: 6V9X [6]) as a principal template, by means of the MODELLER 10.5 software [74]. The modeled region included residues 447–1081, encompassing the pore domain, VSLD, and part of the ankyrin repeats.

Actual template, since some regions of the channel were unresolved in 6V9X, was prepared in a monomeric form as follows. Corresponding regions of the AlphaFold-based model were used to fix gaps in the PDB template (sequence 447–1081 of Q6RI86 was submitted to AlphaFold Server [75], and several regions of output model were added to the MODELLER template: residues 746–767, 796–802, 1011–1038, and 1079). This process resulted in a chimeric scaffold representing a monomer.

This monomeric template was quadrupled and superimposed to the initial 6V9X structure using the *align* procedure in PyMOL, resulting in a “fixed” tetrameric scaffold that was used as input for MODELLER. Homology model of the open rat TRPA1 was further optimized using the PyMOL *sculpting* option to fix potential clashes.

#### 5.6.2. Molecular Dynamics (MD) Simulations

MD simulations were performed using the GROMACS package [76] (versions 2023-2024). Systems were prepared using CHARMM-GUI [77] utilizing *Solution Builder*, *Membrane Builder*, and *Glycan Modeler* modules for aqueous and membrane environments; and N-glycans (added to Asn749 and Asn755 residues). Presence of iodoacetamide at Cys622 residues was considered by including custom YCM residue (constructed using CHARMM-GUI *PDB Manipulator*). All trajectories were calculated using the CHARMM36m [78] force field with the TIP3P water model [79]. The CHARMM36-WYF parameter set [80] was applied to accurately describe cation-π interactions.

MD simulations included the following stages: energy minimization (steepest descent algorithm), NVT equilibration (250 ps), NPT equilibration (for TRPA1-containing systems) with the C-rescale barostat [81] (2000 ps), and production MD (500 ns). The main simulations were conducted in the NPT ensemble at 310 K using the V-rescale thermostat and the Parrinello-Rahman barostat, with a time step of 2 fs. No restraints were applied during the main simulations, allowing the protein, glycans, and lipids to freely relax. Details on systems composition can be found in Table S4.

#### 5.6.3. Clustering of Trajectories and Initial NMR Structures

Clustering of trajectories and structures was performed using the *gromos* method implemented in GROMACS (gmx cluster utility). Conformational clustering of the TRPA1 channel was performed using residues exposed to the extracellular side and accessible for peptide binding (731–784, 815–842, and 885–947) and cutoff of 0.2 nm yielding 131 conformations. Initial NMR structure of HCIQ2c1 with 20 conformations was clustered with the RMSD cutoff of 0.14 nm, yielding 4 conformations. The combined 2000 ns MD trajectory of HCIQ2c1 was clustered with a 0.3 nm cutoff, yielding 49 representative conformations for subsequent docking.

#### 5.6.4. Ensemble Docking

Docking was performed using the MEGADOCK 4.1.1 software [82] with own *bash* automatization scripts, which enabled pairwise search of peptide and channel relative orientations and positions to identify optimal binding modes. Active site of TRPA1 included residues 731–784, 815–842, and 885–947; while residues 447–730, 785–814, 843–884, and 948–1081 were set inactive by *block* procedure. A total of 131 × 49 = 6419 docking runs were conducted, with each run generating 100 solutions, resulting in 641,900 docking solutions.

#### 5.6.5. Docking Analysis and Solution Filtering

Docking solutions were analyzed using PLATINUM [34] and custom *python* scripts to assess ionic interactions, hydrogen bonds, stacking, π–cation, and molecular hydrophobicity potential (MHP) interactions, as well as hydrophobic complementarity (*Cp*) [39] and interface area (*S*). Solutions were filtered based on criteria listed in Table S3. Out of 641,900 solutions, only four passed the filtering criteria and were selected for further MD simulations.

Preparation of tetrameric complexes for selected solutions, structural optimization, and removal of clashes were performed using PyMOL. The *align* procedure was used to prepare starting models of the open TRPA1 channel complexes. For elimination of potential overlaps and unfavorable contacts, PyMOL *sculpting* option was employed.

#### 5.6.6. Analysis of Trajectories

For each peptide in each MD replica, the RMSD of the backbone relative to its position after equilibration was calculated using the *gmx rms* tool in GROMACS to assess binding modes stability. Intermolecular interaction analysis was performed using IMPULSE package [83] and MDAnalysis library [84]. IMPULSE’s *cont_stat.js* tool was used to analyze ionic, ion-dipole contacts, hydrogen bonds, stacking, cation-π, and MHP interactions. Hydrophobic contacts were calculated in MDAnalysis based on the distance ≤ 5 Å between carbon and sulfur atoms in the side chains of hydrophobic residues (Ala, Val, Leu, Ile, Pro, Cys, Met, Phe, Tyr, Trp) and the methyl group of threonine. For each contact, we measured its lifetime as a fraction of the MD trajectory: 0 means that the contact was never observed, 1—the contact was maintained throughout the entire trajectory. As an additional measure of overall complex stability, we also calculated the interface area between HCIQ2c1 and the TRPA1 channel and lipids, using IMPULSE’s *dmdS.mk* tool.

## Figures and Tables

**Table 1:** Long-lived (lifetime > 0.2) intermolecular interactions observed in the MD simulations of the TRPA1/HCIQ2c1 complexes ^1^.

**TRPA1**	**TRPA1/HCIQ2c1 complex**				**TRPA1**	**TRPA1/HCIQ2c1 complex**		
	**1–4**	**2–1**	**5–1**			**1–4**	**2–1**	**5–1**
**VSLD_3_ S1–S2**					**PD_2_ S5–P1**			
K741	**E27^I^ 0.85**	**E27^I^ 0.89**			N896		N4^H^ 0.48	**R14^H^ 0.51**
Q743		S7^H^ 0.31			F897	**P33^B^ 0.56**		R14^P^ 0.39
		S26^H^ 0.21				**I35^BH^ 0.92**		
M746	P33^B^ 0.48	**P9^B^ 0.66**			D899	**R16^I^ 0.66**	R57^I^ 0.35	**K11^I^ 0.93**
I753		**P9^B^ 0.57**	C15^B^ 0.40			**I35^H^ 0.52**		
			**C39^B^ 0.91**			**G37^H^ 0.79**		
I754	**T28^H^ 0.67**				A900	**V12^B^ 0.68**		
N755	**G29^H^ 0.70**		**G40^H^ 0.62**		T903		**F24^B^ 0.64**	**V12^B^ 0.75**
g755 ^2^	R53^H^ 0.29	**G3^H^ 0.58**					S26^H^ 0.31	**I35^B^ 0.76**
E756		I1^I^ 0.22			L906	**V12^B^ 0.90**		**V12^B^ 0.74**
T757	F24^B^ 0.27		**Y36^B^ 0.95**					**F34^B^ 0.87**
	**A58^H^ 0.68**							**I35^B^ 0.72**
I758			R21^H^ 0.24		D918	R14^I^ 0.23		
			**Y36^BH^ 0.93**		**PD_2_ P2–P6**			
S759			E47^H^ 0.48		E933	S18^H^ 0.22		
E762		**K11^I^ 0.68**			A935		I1^B^ 0.77	
E763		V12^H^ 0.43			Y936		I1^B^ 0.79	
		**R14^I^ 0.52**			**PD_1_ P2–P6**			
R764			R16^H^ 0.48		R922			E27^I^ 0.29
			**Y36^P^ 0.85**		D923			K30^I^ 0.27
			**G37^H^ 0.92**		E927	K11^I^ 0.20		**K10^I^ 0.70**
I765		P9^B^ 0.28				R14^I^ 0.23		
		V12^B^ 0.23			F930	V12^B^ 0.31		**F34^B^ 0.58**
N766	E27^H^ 0.20		R14^H^ 0.34					**I35^B^ 0.75**
**VSLD_3_ S3–S4**					R931		A58^I^ 0.27	
A831		I1^B^ 0.45			E933			K30^I^ 0.41
Y832		**I1^B^ 0.80**			**Lipids**			
**Total number of** **interactions**	**20**	**23**	**27**		POPC		K30^I^ 0.43	**R16^I^ 2.25 ^3^**
							T28^B^ 0.41	**G17^H^ 0.81**
**Total lifetime**	**10.4**	**11.3**	**18.9**					

^1^ Along with the interacting residues, lifetimes as a fraction of the whole 500-ns MD trajectory are given. H, I, P, and B denote type of interaction: hydrogen bond, ionic bond, π-cation, and hydrophobic contact, respectively. The interactions with life-time ≥ 0.5 of the MD length are shown in bold. ^2^ g755 denotes glycan group attached to N755. ^3^ The lifetime > 1.0 indicates the presence of several simultaneous interactions of this type.

## Data Availability

Experimental restraints and atomic coordinates for the HCIQ2c1 have been deposited in PDB, under accession code 9IJW. The NMR chemical shifts were deposited in the BMRB database, under accession code 36676.

## Supplementary Materials

The following supporting information can be downloaded at: https://www.mdpi.com/article/10.3390/md22120542/s1, Figure S1: Purification of the ^15^N-HCIQ2c1 peptide; Figure S2: The non-normalized outward current traces for the data presented in Figure 2; Figure S3: The non-normalized current traces for the data presented in Figure 3; Figure S4: 20 best CYANA structures of HCIQ2c1 in aqueous solution; Figure S5: ^15^N relaxation data for HCIQ2c1 and results of the ‘model-free’ analysis; Figure S6: Docking solutions of the TRPA1/HCIQ2c1 complex, close-up top and side views; Figure S7: Characteristics of TRPA1/HCIQ2c1 complexes during 500 ns MD simulations; Figure S8: MD snapshots of the TRPA1/HCIQ2c1 complexes 1–4 and 2–1; Table S1: Open field test. Parameters of motor and orienting-exploratory activity of mice treated by HCIQ2c1; Table S2: Statistics for the best CYANA structures of HCIQ2c1; Table S3: Parameters of the selected docking solutions of the TRPA1/HCIQ2c1 complex; Table S4: Composition of systems used for MD calculations; Table S5: Parameters of the 250–500 ns fragments of TRPA1/HCIQ2c1 MD trajectories.
