# Supplementary material for: Sea Anemone Kunitz Peptide HCIQ2c1: Structure, Modulation of TRPA1 Channel, and Suppression of Nociceptive Reaction In Vivo
Source: Mar Drugs. 2024 Dec 2;22(12):542. doi: 10.3390/md22120542 (PMC11678340; doi:10.3390/md22120542)
Supplement: Supplementary file 1 [file marinedrugs-22-00542-s001.zip › marinedrugs-3294120-supplementary.pdf]

Supplementary Materials

Sea anemone Kunitz-type peptide modulates TRPA1 channel and suppresses a nociceptive reaction *in vivo*

Table S1. Open field test. Parameters of motor and orienting-exploratory activity of mice treated by HCIQ2c1.

Groups	Central time, s	Border time, s	Activity time, s	Passivity time, s	Mean speed, m/s	Distance travelled, m	# Rearing up	# Holes explored
**Saline**	45.1±12.3	134.3±12.9	134.2±15.4	43.2±14.2	0.17±0.03	29.8±5.6	20.9±5.9	7.3±3.6
**HCIQ2c1 1 mg/kg**	43.6±8.8	134.7±10.1	131.4±6.7	44.5±10.0	0.16±0.02	27.9±3.0	17.0±5.3	10.6±2.9
**HCIQ2c1 0.1 mg/kg**	43.8±11.7	135.2±11.7	131.2±13.3	46.2±13.1	0.17±0.02	29.2±4.1	17.7±6.2	10.0±2.2
**HCIQ2c1 0.01 mg/kg**	48.9±16.2	129.2±14.3	129.2±10.4	46.5±9.4	0.16±0.02	28.8±3.9	14.2±5.0	8.5±1.6

Notes: Central time — time spent on the central zone; Border time — time spent on the border zone; Activity time – time of mouse activity; Passivity time – time of mouse immobility; Mean speed — average travel speed; Distance traveled – distance traveled by animals for 3 minutes; # Rearing up — vertical activity, the number of racks; # Holes explored – the number of peeps into minks. Control animals received the same volume of saline. Results are presented as mean ± S.D. (n = 7). The data groups were compared with control (saline) group using one-way ANOVA/Dunnett’s multiple comparisons test.

Table S2. Statistics for the best CYANA structures of HCIQ2c1.

Distance and angle restraints	
Total NOE contacts	440
Intraresidual	95
Sequential (∣i – j∣ = 1)	149
Medium range (1 < ∣i – j∣ ≤ 4)	57
Long range (∣i – j∣ > 4)	139
Hydrogen bond restraints (bonds/upper/lower)	31/62/62
S–S bond restraints (bonds/upper/lower)	3/9/9
Torsion angle restraints	81
Angle φ	54
Angle χ1	27
Total restraints/per residue	663/11.4

Statistics for the calculated structures	
Structures calculated/selected	200/20
CYANA target function (Å^2)	1.49 ± 0.07
Violations of restraints	
Distance (> 0.2 Å)	0
van der Waals (> 0.2 Å)	4
Dihedral angles (> 1°)	0
RMSD (Å) all residues (1-58)	
Backbone	0.61 ± 0.11
Heavy atoms	1.30 ± 0.14
R.M.S.D. (Å) secondary structure (19-36, 45-57)	
Backbone	0.30 ± 0.07
Heavy atoms	0.94 ± 0.11

RMSD – Root mean square deviation.

Table S3. Parameters of the selected docking solutions of the TRPA1/HCIQ2c1 complex.

**Docking solution**	**S, contact area, nm^2**	***Cp*, complementarity of polar and hydrophobic properties**	**# Classic interactions (Ion, H-bond, π-Cat, Stack)**	**# Ionic interactions, total**	**# Ionic interactions with VSLD**	**# Ionic interactions with PD**	**# Hydrophobic contacts**
1	21.2	0.65	19	6	2	4	3
2	21.4	0.62	13	6	3	3	3
3	20.7	0.62	13	6	4	2	3
4	21.4	0.71	18	10	2	8	4
Criteria	>20.0	>0.55	≥13	≥6			

Table S4. Composition of systems used for MD calculations.

Docking solution – MD trajectory	System [1]	Box dimensions [2], nm^3	#atoms	MD length, ns
**HCIQ2c1 peptide in aqueous solution**				
	$HCIQ2c1/Water_{6061}/Na^{+}_{17}/Cl^{-}_{21}$	5.74×5.74×5.74	19 080	500
	$HCIQ2c1/Water_{6052}/Na^{+}_{17}/Cl^{-}_{21}$	5.73×5.73×5.73	19 053	
	$HCIQ2c1/Water_{6402}/Na^{+}_{18}/Cl^{-}_{22}$	5.86×5.86×5.86	20 105	
	$HCIQ2c1/Water_{5478}/Na^{+}_{15}/Cl^{-}_{19}$	5.55×5.55×5.55	17 327	
**Rat TRPA1 in POPC bilayer in aqueous solution**				
	$TRPA1/POPC_{512}/Water_{96540}/Na^{+}_{264}/Cl^{-}_{288}$	14.53×14.53×18.67	401 564	600
**Complex of rat TRPA1 with HCIQ2c1 in POPC bilayer in aqueous solution**				
1–1	$TRPA1/HCIQ2c1_{4}/$ $POPC_{510}/Water_{103318}/Na^{+}_{282}/Cl^{-}_{314}$	14.40×14.40×20.04	425 106	500
2–2	$TRPA1/ HCIQ2c1_{4}/$ $POPC_{500}/Water_{96705}/Na^{+}_{265}/Cl^{-}_{297}$	14.27×14.27×19.42	403 893	
3–3	$TRPA1/ HCIQ2c1_{2}/$ $POPC_{510}/Water_{95212}/Na^{+}_{260}/Cl^{-}_{284}$	14.43×14.43×18.70	399 018	
4–4 [3]	$TRPA1/ HCIQ2c1/$ $POPC_{511}/Water_{99434}/Na^{+}_{273}/Cl^{-}_{293}$ (*)	14.59×14.59×18.80	410 981	
4–5 [3]	$TRPA1/ HCIQ2c1/$ $POPC_{511}/Water_{99434}/Na^{+}_{273}/Cl^{-}_{293}$ (*)	14.62×14.62×18.80		

[1] POPC — 1-palmitoyl-2-oleoyl-sn-glycero-3-phosphocholine.

Na^+ and Cl^- ions were used to neutralize the protein and to approximate physiological solution with [NaCl] = 0.15 M.

[2] The dimensions of box for the last frame of production MD run are indicated.

[3] In case of 4th docking solution, two replicas with independent velocities generation were calculated.

Table S5. Parameters of the 250–500 ns fragments of TRPA1/HCIQ2c1 MD trajectories [1].

Docking solution – MD trajectory – peptide	RMSD ± SD, nm	S peptide / channel ± SD, nm^2	S peptide / lipids ± SD, nm^2	# Classic interactions (Ion, H-bond, π-Cat, Stack)	# Hydrophobic contacts	# Classic + Hydrophobic interactions
1–1–1	1.0 ± 0.2	21.1 ± 1.7	3.0 ± 1.5	9.7 ± 2.6	6.8 ± 1.4	16.5 ± 3.2
1–1–2	1.1 ± 0.1	24.3 ± 3.3	2.2 ± 1.3	9.3 ± 3.0	7.2 ± 1.4	16.5 ± 3.3
1–1–3	1.5 ± 0.2	17.4 ± 2.6	2.5 ± 1.8	6.2 ± 2.2	6.8 ± 1.9	13.0 ± 3.2
**1–1–4**	**1.0 ± 0.1**	**26.9 ± 1.7**	**1.5 ± 0.6**	**12.8 ± 2.5**	**6.6 ± 1.6**	**19.4 ± 2.9**
mean	1.1	22.4	2.3	9.5	6.8	16.4
**2–2–1** [2]	**1.0 ± 0.2**	**24.7 ± 2.1**	**4.6 ± 1.3**	**12.9 ± 3.0**	**7.4 ± 1.4**	**20.3 ± 3.8**
2–2–2 [2]	3.1 ± 0.2	7.5 ± 1.6	0.0 ± 0.1	3.8 ± 2.2	0.1 ± 0.3	3.8 ± 2.3
2–2–3 [2]	3.2 ± 0.3	4.7 ± 2.0	0.1 ± 0.5	3.4 ± 1.8	0.1 ± 0.5	3.5 ± 1.9
2–2–4	0.6 ± 0.1	24.7 ± 2.2	5.5 ± 2.9	10.9 ± 2.5	3.7 ± 1.4	14.6 ± 3.2
mean	2.0	15.4	2.5	7.7	2.8	10.6
3–3–1	1.5 ± 0.3	20.0 ± 3.5	2.9 ± 1.8	9.8 ± 3.5	2.6 ± 2.0	12.4 ± 3.5
3–3–2	2.1 ± 0.4	12.5 ± 3.8	0.7 ± 1.0	6.3 ± 3.1	4.3 ± 2.0	10.7 ± 4.2
mean	1.8	16.3	1.8	8.0	3.5	11.5
4–4–1	1.0 ± 0.3	13.2 ± 1.9	3.3 ± 0.5	6.2 ± 1.7	4.8 ± 1.4	11.0 ± 2.3
**4–5–1**	**1.0 ± 0.1**	**21.7 ± 1.8**	**7.1 ± 1.3**	**13.1 ± 2.3**	**8.4 ± 1.6**	**21.6 ± 2.9**
mean	1.0	17.5	5.2	9.7	6.6	16.3
Criteria	≤ 1.0	≥ 20.0		≥ 12.0	≥ 6.0	≥ 18.0

[1] TRPA1/HCIQ2c1 complexes selected for strong intramolecular interactions and high stability are shown in bold. [2] These three peptide molecules came into contact with each other in the middle of the MD trajectory. So, the corresponding complexes should be considered with caution.

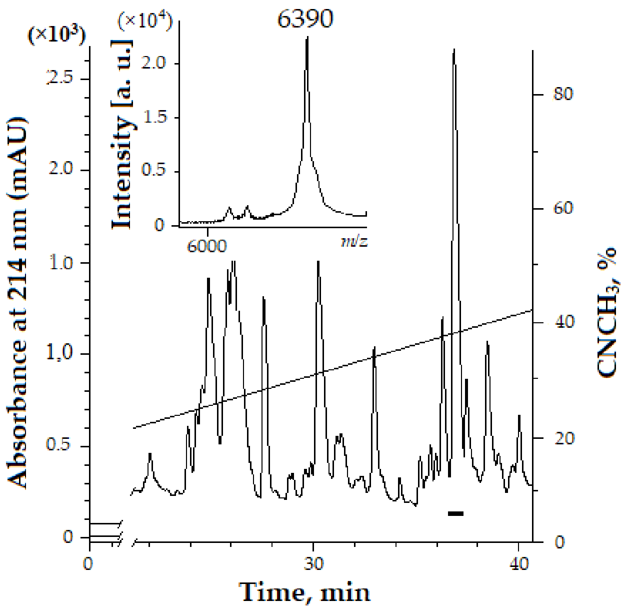

Figure S1. Purification of the ^{15}N-HCIQ2c1 peptide. HPLC elution profile of ^{15}N-HCIQ2c1 on reverse-phase column Jupiter C4 (250 × 10 mm), using a linear gradient of CH_3CN concentration (0–70% in 70 min) with 0.1% TFA and flow rate of 2 mL/min. Insert: MALDI-TOF/MS spectrum of ^{15}N-HCIQ2c1.

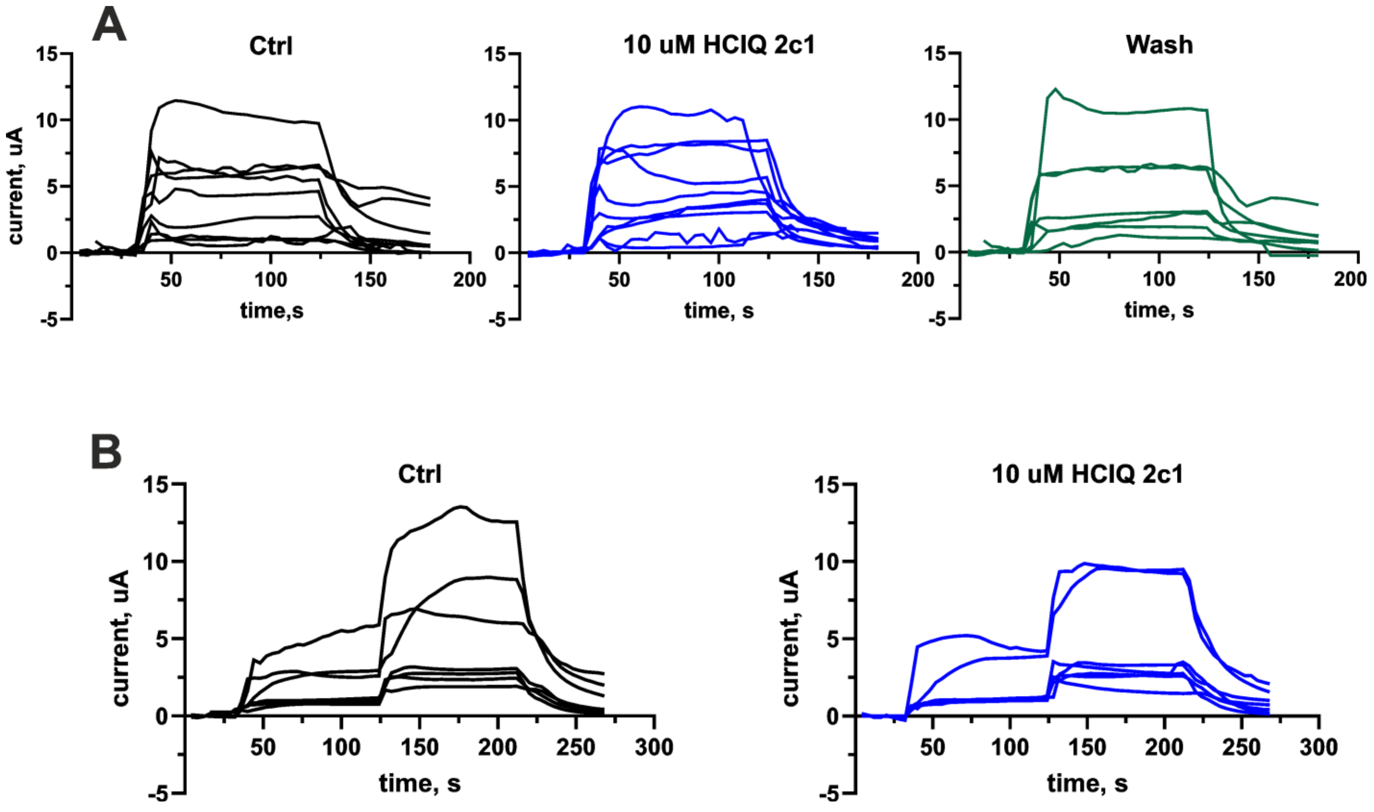

Figure S2. The non-normalized outward current traces for the data presented in Figure 2. The diclofenac-evoked currents in *X. laevis* oocytes expressing rat TRPA1 are shown. See Figure 2 in the main manuscript for details.

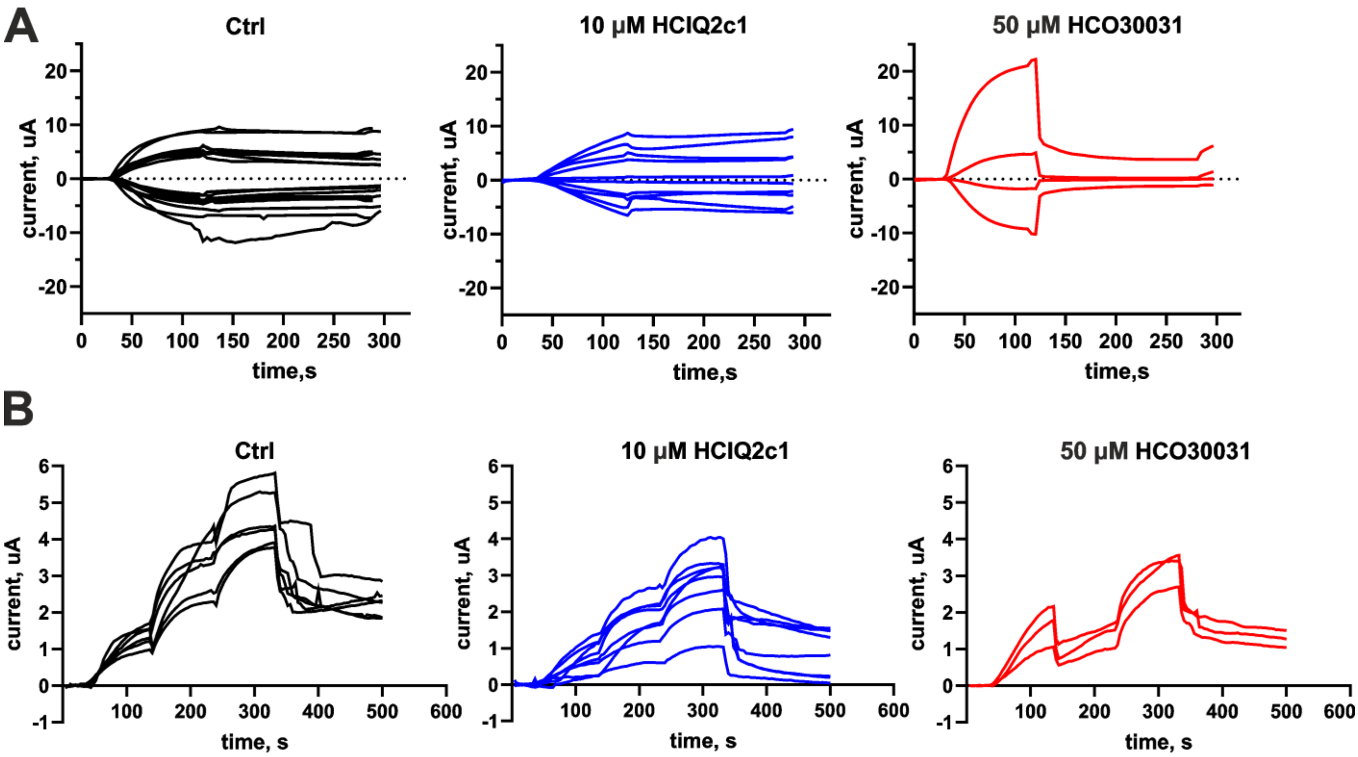

Figure S3. The non-normalized current traces for the data presented in Figure 3. The AITC-evoked currents in *X. laevis* oocytes expressing rat TRPA1 are shown. See Figure 3 in the main manuscript for details.

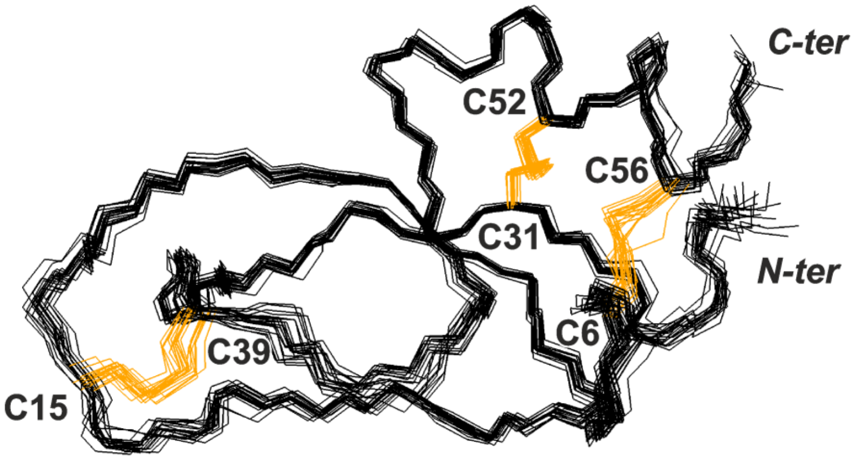

Figure S4. 20 best CYANA structures of HCIQ2c1 in aqueous solution.

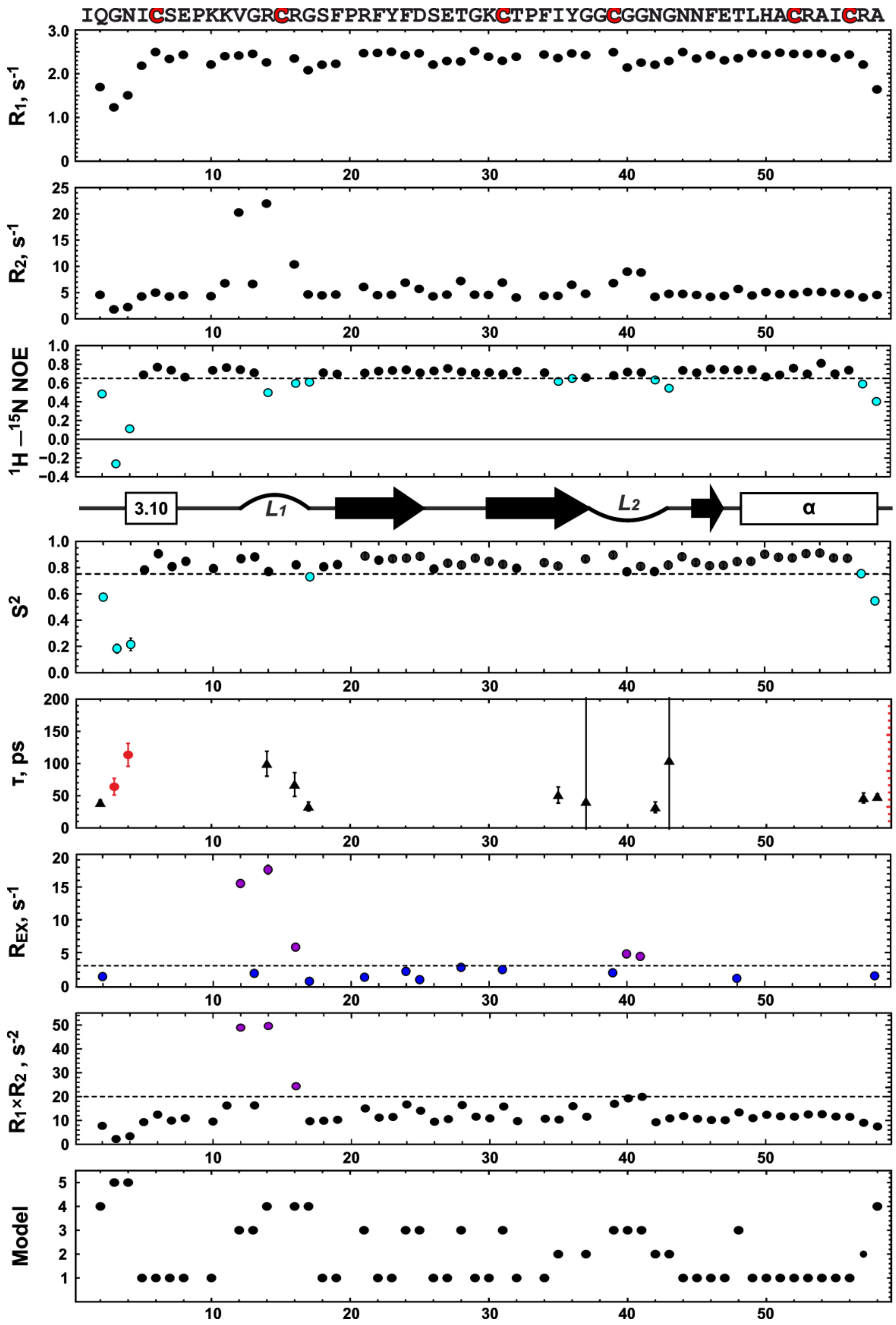

Figure S5. ^{15}N relaxation data (R_1, R_2, NOE) for HCIQ2c1 (60 MHz, pH 4.5, 30°C) and results of the 'model-free' analysis (S^2, τ_e, and R_{EX}) describing dynamics of the peptide backbone in aqueous solution. R_1 and R_2 are the values of longitudinal and transverse ^{15}N relaxation rates, respectively. Heteronuclear ^{15}N–{^{1}H} NOE values and generalized-order parameters (S^2) revealed residues with high-amplitude mobility on the ps–ns time-scale (where $S^2 < 0.75$ or ^{15}N–{^{1}H} NOE). τ_e – effective correlation times for ps and ns internal motions. Exchange contributions to the R_2 relaxation rates of ^{15}N nuclei (R_{EX}) and the product of relaxation rates $R_1 \times R_2$ revealed residues with the mobility on the μs–ms time-scale (where $R_{EX} \geq 3.0$ s^{-1} or $R_1 \times R_2 > 20.0$ s^{-2}, and $3 \geq R_{EX} > 0$ s^{-1}). Model is the number of relaxation model assigned by the FastModelFree software.

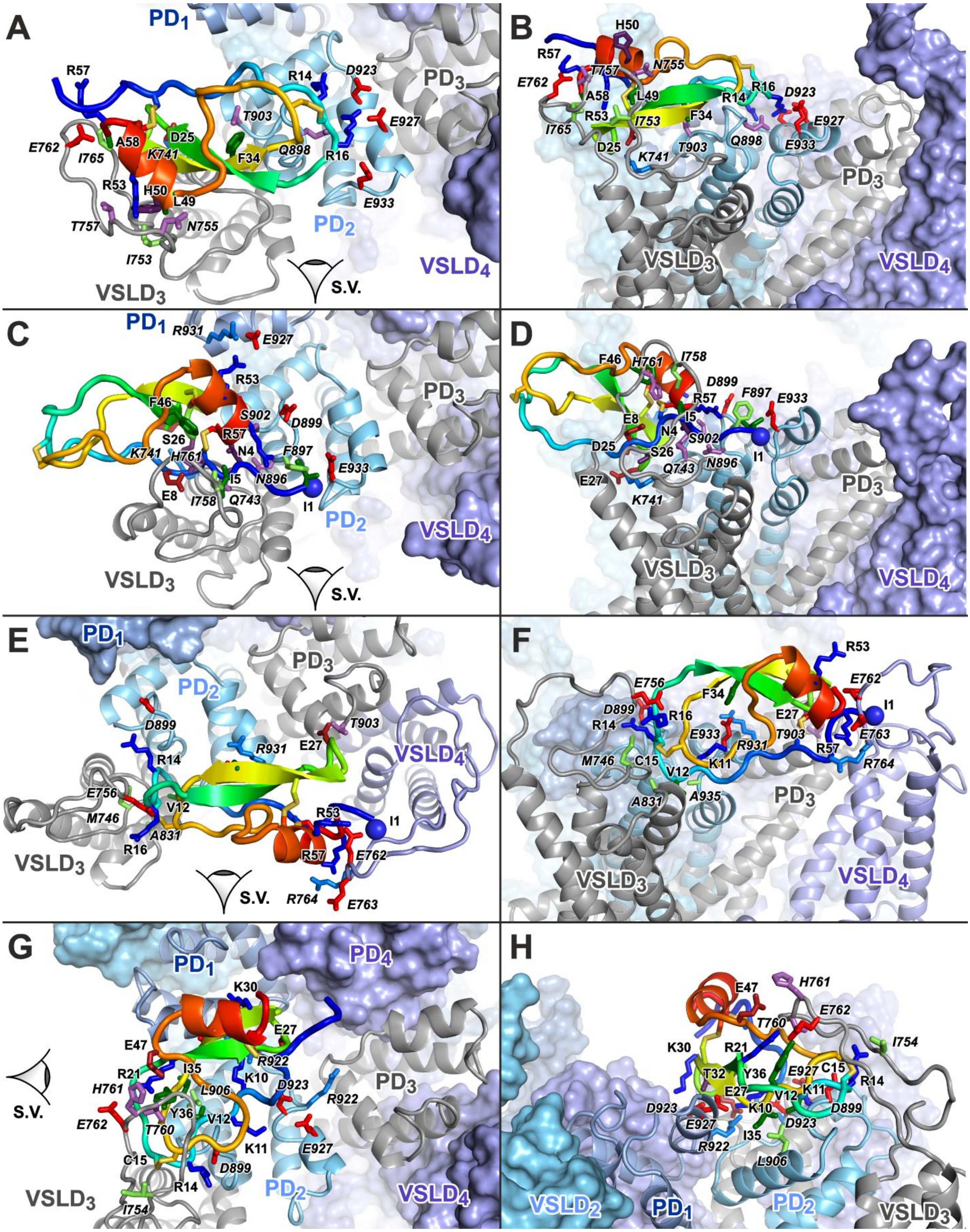

Figure S6. Docking solutions of the TRPA1/HCIQ2c1 complex, close-up top (**A**,**C**,**E**,**G**) and side (**B**,**D**,**F**,**H**) views. Solution #1 (**A**,**B**); #2 (**C**,**D**); #3 (**E**,**F**); #4 (**G**,**H**). The directions of side-views are indicated by eye symbol on the corresponding top view panels. Colors and designations are as in Figures 11 and 12. The N-glycan groups attached to VSLDs were omitted in the docking calculations.

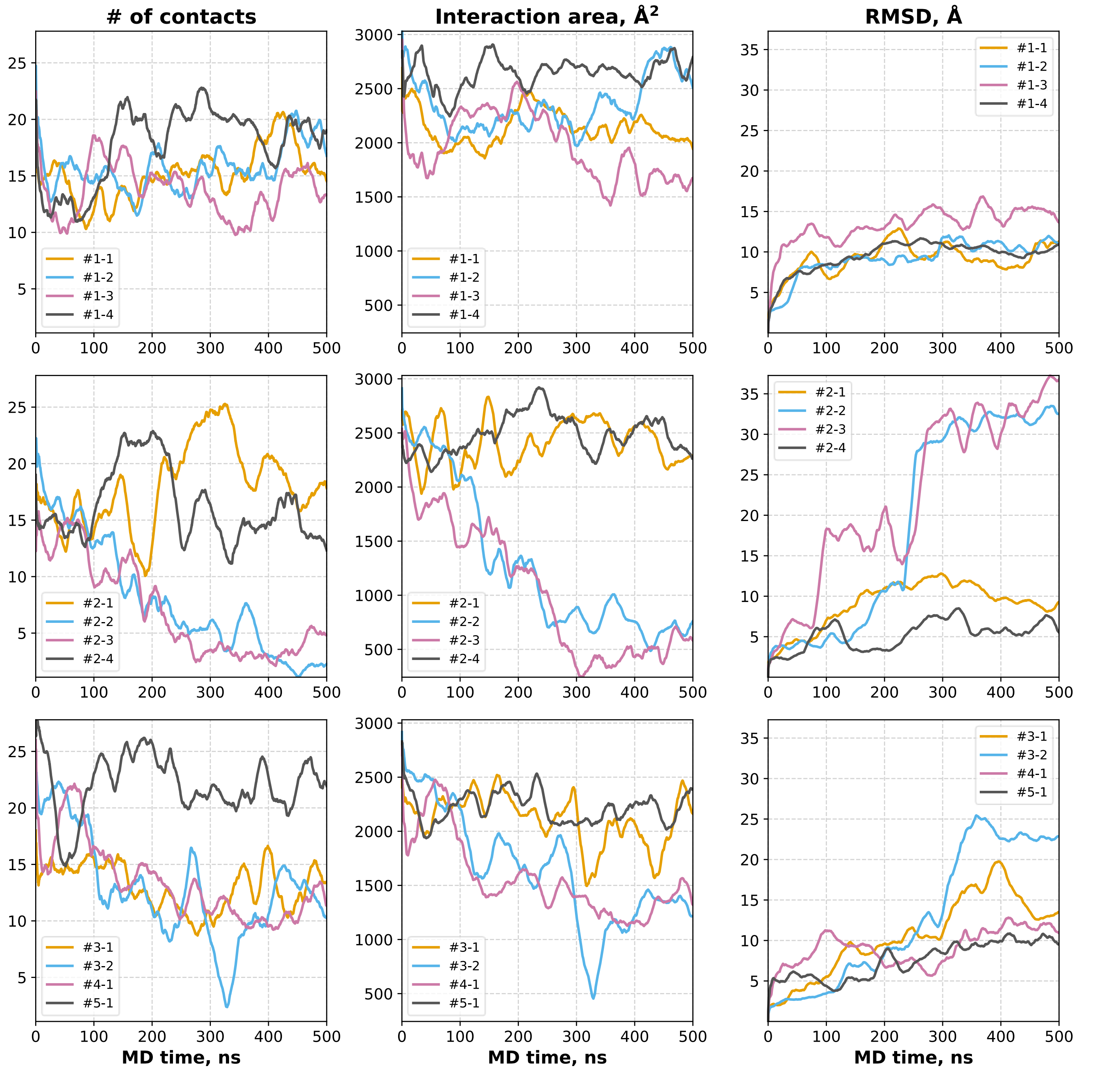

Figure S7. Characteristics of TRPA1/HCIQ2c1 complexes during 500 ns MD simulations. Each complex is labelled by two numbers: #**a-b**, where *a* denotes the MD replica number (from 1 to 5), and *b* is the toxin number within the MD replica (from 1 to 4). In replicas 1 and 2, the systems contained four toxins each; in replica 3 – two toxins; and in replicas 4 and 5 – one toxin each. MD replicas ##1–3 correspond to docking solutions ##1–3, while the replicas 4 and 5 were both based on the docking solution #4, but use independent initial velocity generation. (**Left column**) – total number of intermolecular interactions stabilizing the complex. The number of ionic contacts, hydrogen bonds, stacking interactions, cation–π interactions, and hydrophobic contacts were calculated at each time-point. The hydrophobic contacts are defined by a 5 Å distance cutoff between C/S atoms of hydrophobic/aromatic amino acid side chains. (**Middle column**) TRPA1–HCIQ2c1 contact area in the complex (Å^2). (**Right column**) – the root-mean-square deviation (RMSD) of the toxin relative to it initial position after minimization and equilibration. All data are overaged in 50 ns window.

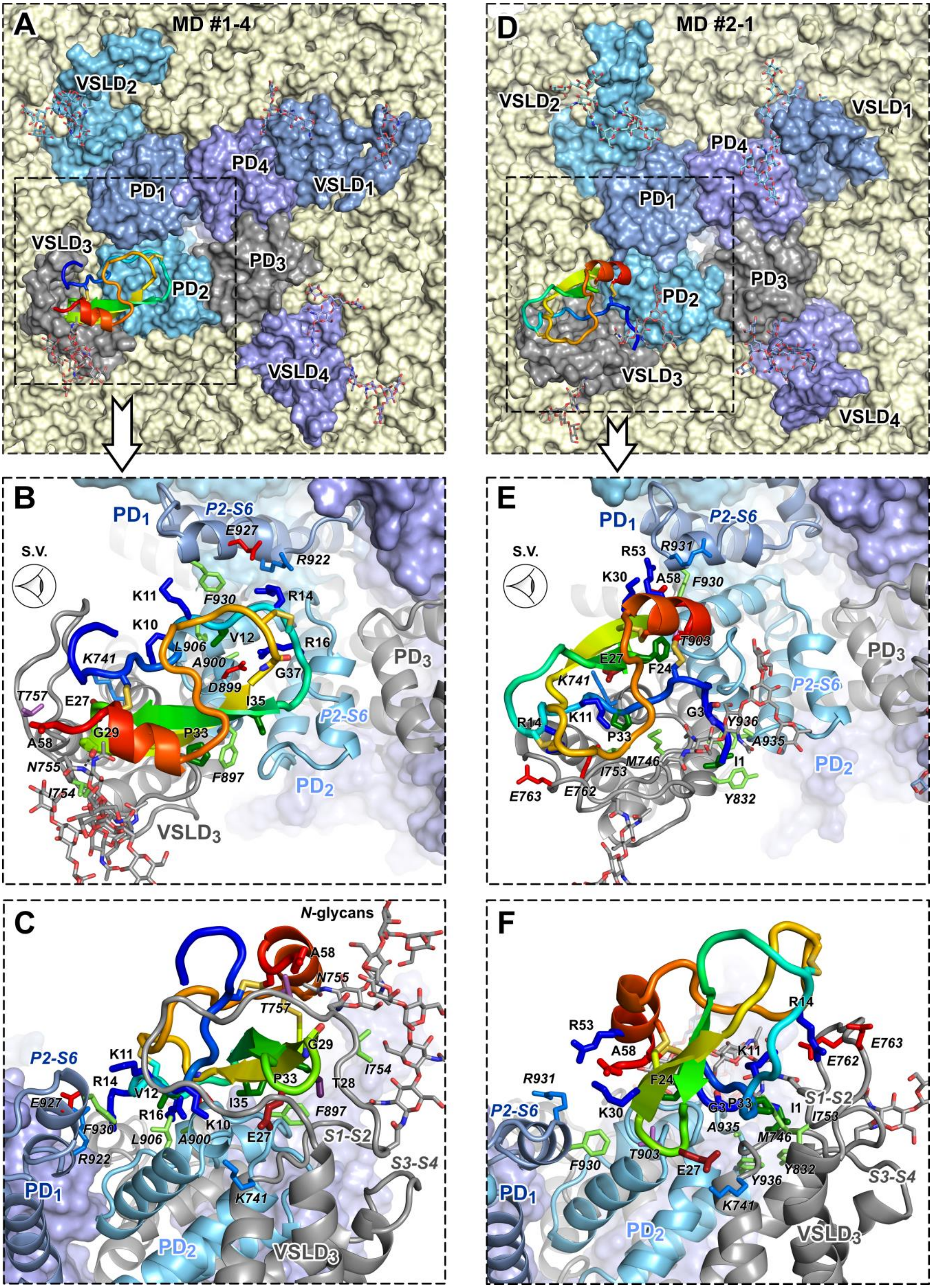

Figure S8. MD snapshots of the TRPA1/HCIQ2c1 complexes 1–4 (**A,B,C**) and 2–1 (**D, E, F**) (see Tables 3 and 4). Colors and designations are as in Figures 11 and 12. (**A,D**) Top view on the simulation system. (**B,E**) Close-up top and (**C,F**) side views of the TRPA1/HCIQ2c1 complex. The directions of side-views are indicated by eye symbol on the corresponding top view panels.
